# RNF168 cooperates with RNF8 to mediate FOXM1 ubiquitination and degradation in breast cancer epirubicin treatment

**DOI:** 10.1038/oncsis.2016.57

**Published:** 2016-08-15

**Authors:** M Kongsema, S Zona, U Karunarathna, E Cabrera, E P S Man, S Yao, A Shibakawa, U-S Khoo, R H Medema, R Freire, E W-F Lam

**Affiliations:** 1 Department of Surgery and Cancer, Imperial College London, London, UK; 2 Unidad de Investigación, Hospital Universitario de Canarias, Instituto de Tecnologías Biomédicas, Ofra s/n, La Laguna, Tenerife, Spain; 3 Department of Pathology, Li Ka Shing Faculty of Medicine, The University of Hong Kong, Hong Kong SAR, China; 4 Division of Cell Biology, The Netherlands Cancer Institute, Amsterdam, The Netherlands

## Abstract

The forkhead box M1 (FOXM1) transcription factor has a central role in genotoxic agent response in breast cancer. FOXM1 is regulated at the post-translational level upon DNA damage, but the key mechanism involved remained enigmatic. RNF168 is a ubiquitination E3-ligase involved in DNA damage response. Western blot and gene promoter-reporter analyses showed that the expression level and transcriptional activity of FOXM1 reduced upon RNF168 overexpression and increased with RNF168 depletion by siRNA, suggesting that RNF168 negatively regulates FOXM1 expression. Co-immunoprecipitation studies in MCF-7 cells revealed that RNF168 interacted with FOXM1 and that upon epirubicin treatment FOXM1 downregulation was associated with an increase in RNF168 binding and conjugation to the protein degradation-associated K48-linked polyubiquitin chains. Consistently, RNF168 overexpression resulted in an increase in turnover of FOXM1 in MCF-7 cells treated with the protein synthesis inhibitor cycloheximide. Conversely, RNF168, knockdown significantly enhanced the half-life of FOXM1 in both absence and presence of epirubicin. Using a SUMOylation-defective FOXM1-5x(K>R) mutant, we demonstrated that SUMOylation is required for the recruitment of RNF168 to mediate FOXM1 degradation. In addition, clonogenic assays also showed that RNF168 mediates epirubicin action through targeting FOXM1, as RNF168 could synergise with epirubicin to repress clonal formation in wild-type but not in FOXM1-deficient mouse embryo fibroblasts (MEFs). The physiological relevance of RNF168-mediated FOXM1 repression is further emphasized by the significant inverse correlation between FOXM1 and RNF168 expression in breast cancer patient samples. Moreover, we also obtained evidence that RNF8 recruits RNF168 to FOXM1 upon epirubicin treatment and cooperates with RNF168 to catalyse FOXM1 ubiquitination and degradation. Collectively, these data suggest that RNF168 cooperates with RNF8 to mediate the ubiquitination and degradation of SUMOylated FOXM1 in breast cancer genotoxic response.

## Introduction

Breast cancer is one of the leading causes of death in women worldwide. Anthracyclines, platinum compounds, methylating agents and ionizing irradiation are common genotoxic anticancer agents employed for the treatment of breast cancer patients not suitable for endocrine therapy as well as those with secondary or metastatic diseases. Moreover, these DNA-damaging agents are used after primary treatments, such as surgery or radiation, to prevent cancer recurrence. However, patients invariably develop resistance to these genotoxic agents, resulting in ineffective treatment, cancer progression and disease relapse.^1^ The insurgence of genotoxic agent resistance is primarily mediated through cellular DNA damage response (DDR). DDR involves the initiation of DNA damage repair, the activation of cell-cycle checkpoint and the induction of apoptosis or senescence, which ultimately influence sensitivity to genotoxic chemotherapy and cell fate.

The Forkhead box M1 (FOXM1) transcription factor has a pivotal role in promoting cell proliferation, migration, invasion, angiogenesis, stem cell renewal and DNA damage repair, thus influencing cancer initiation, progression, metastasis, angiogenesis and drug sensitivity. Recent research also indicates that deregulated FOXM1 overexpression confers genotoxic and other cancer chemotherapeutic agent resistance.^2, 3, 4, 5, 6, 7^ There is already ample evidence, indicating that FOXM1 acts as a mediator of DDR and a modulator of genotoxic agent sensitivity, through regulating the expression of genes, including BRIP1, NBS1, EXO1, XRCC1, RAD51 and RFC4, involved in DDR.^4, 5, 8, 9, 10^ Despite the importance of FOXM1 in DNA-damaging agent response, the exact mechanisms by which FOXM1 is regulated by genotoxic agents remain a crucial unresolved issue.

Ubiquitination is a post-translational modification whereby ubiquitins (Ubs) become covalently conjugated to target proteins. This process has a key role in regulating cellular protein stability, activity, interaction and localization. Ubiquitination is a three-step enzymatic process, involving ubiquitin activation, conjugation and ligation, mediated by ubiquitin-activating enzymes (E1s), ubiquitin-conjugating enzymes (E2s) and ubiquitin ligases (E3s), respectively. The E3-ubiquitin ligases recognize the specific target proteins to be ubiquitinated and therefore also dictate substrate specificity.^11^ K(Lys)48- and K(Lys)63-linked polyubiquitination polymers are the predominantly ubiquitin chains found on cellular proteins. While proteins with K48-linked ubiquitin polymers are commonly targeted for degradation by the proteasome, K63-linked ubiquitin chains are usually associated with other protein regulatory functions, such as modulating the activity, function and subcellular location of the target proteins or altering protein–protein interactions.

Epirubicin is an anthracycline commonly used for the treatment of breast cancer.^12^ Upon epirubicin treatment, FOXM1 is primarily downregulated at the post-translational level in breast cancer cells.^8, 13, 14^ In line with this, we have recently shown that in response to epirubicin treatment, FOXM1 is modified through SUMOylation, which leads to its ubiquitination and degradation via the proteasome proteolytic pathway.^15^ A novel subgroup of E3-ligases called SUMO-targeted Ubiquitin E3-ligases (STUbLs) provide a link for cross-talks between the SUMO and ubiquitin pathways. Of these STUbLs, a subset, including RNF4 and -8, has been shown to have a specific role in double-strand break (DSB) DDR.^11, 16, 17, 18, 19^ Our proteomic analysis has also identified another related DDR Ubiquitin E3-ligase RNF168 as a putative FOXM1-interacting protein. These ubiquitin E3-ligases are found primarily in the nucleus and their known DDR functions are also identified in the nucleus.^20^ On the basis of these observations, we speculated that these ubiquitin E3-ligases may modulate FOXM1 expression in breast cancer cells and in response to the genotoxic agent epirubicin. To establish a connection between epirubicin-induced FOXM1 SUMOylation and its subsequent targeting to the proteasome for degradation, we sought to identify the ubiquitin E3-ligase(s) involved. We established in this study the ubiquitin E3-ligases RNF8 and RNF168 as the downstream effectors that collectively recognize and mediate the functional consequences of FOXM1 SUMOylation in response to epirubicin treatment.

## Results

### RNF168 downregulates FOXM1 expression in MCF-7 breast cancer cells

**Figure 1:**
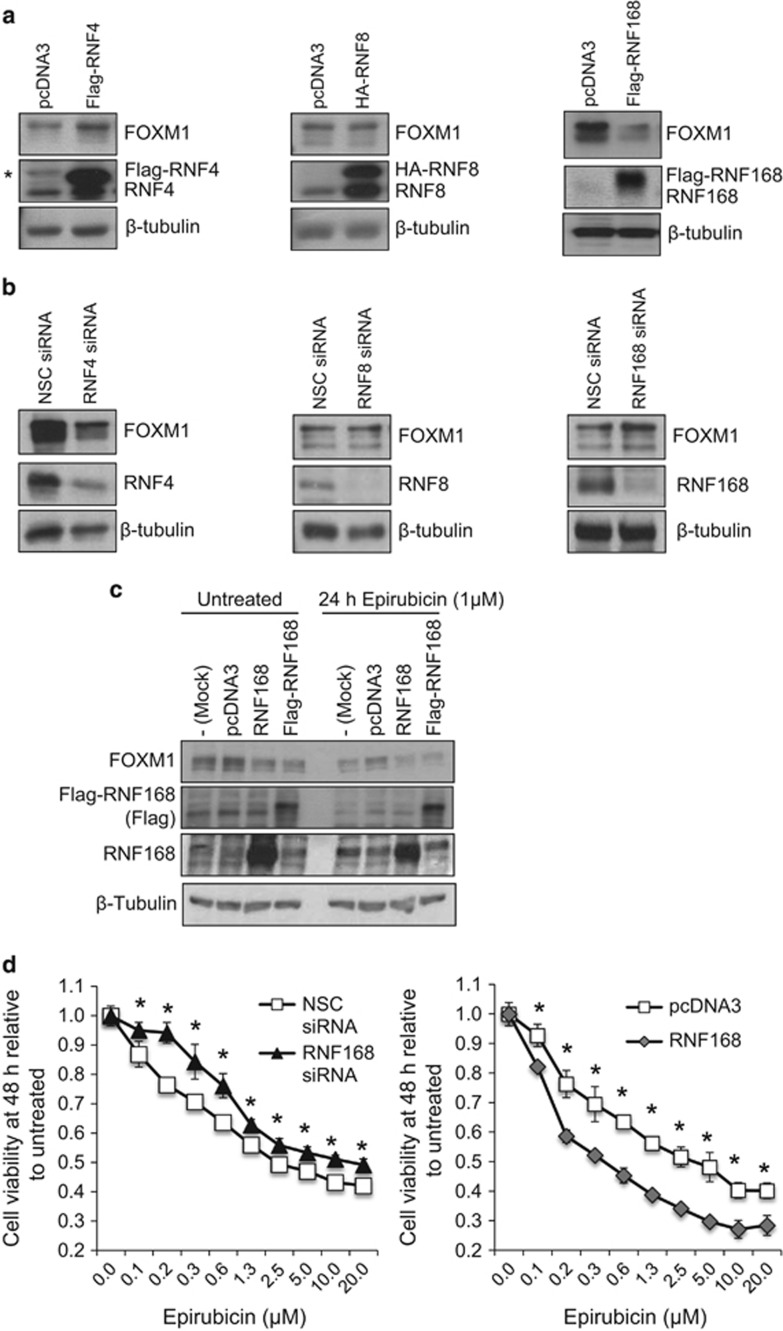
RNF168 negatively regulates FOXM1 expression and cell proliferation in MCF-7 cells. (**a**) Western blot analysis was performed on MCF-7 cells transfected with empty expression vector (pcDNA3) and Flag-RNF4, HA-RNF8 or Flag-RNF168 expression vector for 48 h. The protein expression levels of FOXM1, the RNF E3-ligase and β-tubulin were assessed by western blot analysis (asterisk indicates the non-specific protein band). FOXM1 expression was observed to be downregulated by RNF168, but not by RNF4 and RNF8, at the protein level. (**b**) MCF-7 cells were transfected with NSC siRNA, or with RNF4, RNF8 or RNF168 specific siRNA pool for 48 h. The protein expression levels of FOXM1, the RNF E3-ligase and β-tubulin were assessed by western blot analysis. FOXM1 expression was observed to be upregulated by the silencing of RNF168, but not RNF4 and RNF8, at the protein level. (**c**) MCF-7 cells were either untransfected (Mock), or transfected with pcDNA3-empty vector, with RNF168 or with Flag-RNF168 for 48 h. Western blot analysis was performed on the transfected MCF-7 cells with or without 1 μm epirubicin treatment for 24 h. The protein expression levels of FOXM1, RNF168, Flag-(RNF168) and β-tubulin were assessed by western blot analysis. (**d**) MCF-7 cells were transfected with NSC siRNA, siRNA pool targeting RNF168, pcDNA3-empty vector or pcDNA3-Flag-RNF168. Twenty-four hours after transfection, aliquots of the transfected cells were split into 96-well plates with concentrations of epirubicin indicated and their proliferation analysed at 48 h by SRB assays. Cell proliferation assays revealed that Flag-RNF168 overexpression increased and RNF168 depletion with siRNA decreased epirubicin sensitivity in MCF-7 cells. The results presented as bars representing mean±s.d. of three independent experiments in triplicates. The differences between that RNF168 overexpression or depletion and their relative controls are significant at *P<0.05 levels, except for those untreated.

Our previous proteomic study identified the E3-Ubiquitin ligase RNF168 as a potential FOXM1-interacting protein. Given the role of RNF168 as an E3-Ubiquitin ligase and the involvement of ubiquitination in the epirubicin-induced FOXM1 downregulation,^14, 15^ we hypothesized that RNF168 binds FOXM1 and modulates its expression through the ubiquitin-proteasome degradation pathway. To explore this possibility, we first overexpressed RNF168 and two other DDR-related E3-ligases, RNF4 and RNF8, in the MCF-7 breast carcinoma cells, and studied their effects on FOXM1 expression (Figure 1a). We found that while RNF168 decreased and RNF4 increased FOXM1 expression, RNF8 expression had little effect on the expression levels of FOXM1. Conversely, depletion of RNF4 using siRNA repressed FOXM1 expression, whereas RNF168 knockdown induced FOXM1 accumulation in MCF-7 cells (Figure 1b). Once again, RNF8 silencing had little effect on FOXM1 expression in untreated MCF-7 cells. Together, these data suggest that RNF168, and not RNF4, is a key negative regulator of FOXM1 expression in untreated MCF-7 cells. To confirm this further, we overexpressed either an untagged or a Flag-tagged RNF168 in MCF-7 cells (Figure 1c). Consistently, the results showed that overexpression of RNF168 effectively downregulated FOXM1 in the absence and presence of epirubicin treatment. These results suggest that RNF168 can downregulate FOXM1 expression and might mediate the anti-proliferative effects of epirubicin. To test this idea, we overexpressed and depleted RNF168 in MCF-7 cells and assessed their proliferation in response to different doses of epirubicin. In agreement, the results indicated that RNF168 silencing decreased the anti-proliferative function of epirubicin, while RNF168 overexpression sensitized MCF-7 cells towards epirubicin (Figure 1d).

### RNF168 represses FOXM1 activity in MCF-7 breast cancer cells

**Figure 2:**
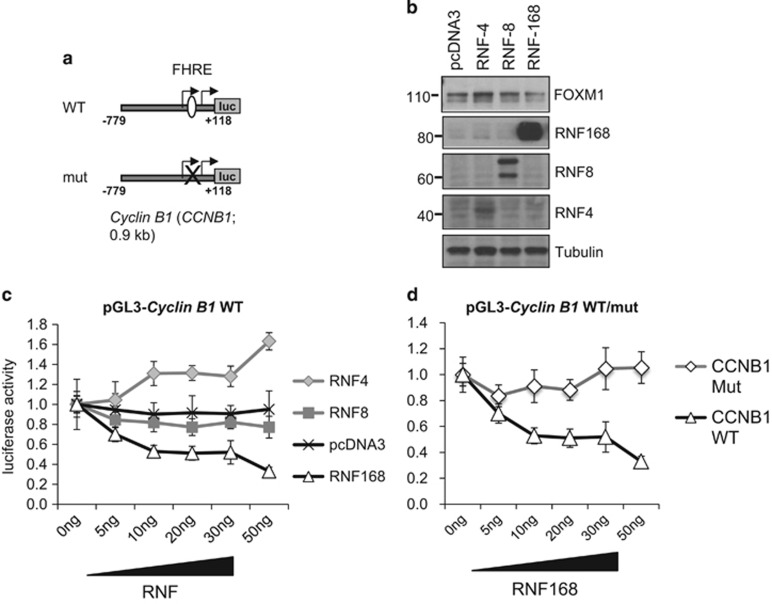
Overexpression of RNF168, but not RNF4 and RNF8, effectively repressed FOXM1 transcriptional activity. (**a**) Schematic showing of the WT (pGL2-*Cyclin B1*-WT) and the FOXM1-mutant (pGL2-*Cyclin B1*-mut) *Cyclin B1* promoter/reporter. (**b**) MCF-7 cells were co-transfected with the WT *cyclin B1* promoter (pGL2-*Cyclin B1*-WT) and 20 ng of the empty expression vector (pcDNA3), Flag-RNF4, HA-RNF8 or Flag-RNF168 expression vector. Forty-eight hours after transfection, the transfected cells were western blotted for the expression of FOXM1, RNF168, RBF8, RNF4 and β-tubulin. (**c**) MCF-7 cells were co-transfected with the luciferase reporter driven by the WT cyclin B1 promoter (pGL2-*Cyclin B1*-WT) and the empty expression vector (pcDNA3) or the Flag-RNF4, HA-RNF8 or Flag-RNF168 expression vector (0–50 ng) for 48 h as in (**b**). Reporter gene activity was expressed as a ratio of firefly luciferase activity to control *Renilla* luciferase activity. The promoter activity assay results are presented as bars representing mean±s.d. of three independent experiments in triplicates. All DNA concentrations were normalized using empty vector. (**d**) MCF-7 cells were co-transfected with Flag-RNF168 expression vector (0, 5, 10, 20, 30 and 50 ng) and the luciferase reporter driven by the WT *cyclin B1* promoter (pGL2-*Cyclin B1*-WT) or the mutant *cyclin B1* promoter (pGL2-*Cyclin B1*-mut) and the Flag-RNF168 expression vector (0–50 ng) for 48 h. Reporter gene activity was expressed as a ratio of firefly luciferase activity to control *Renilla* luciferase activity. The promoter activity assay results are presented as bars representing mean±s.d. of three independent experiments in triplicates. **P⩽0.01, ***P⩽0.001 and ns indicates no significance by Student's *t*-test.

To further confirm our results, we next investigated the effects of overexpression of RNF4, RNF8 and RNF168 on FOXM1 activity using the FOXM1-regulated *cyclin B1* promoter as a reporter. Consistent with our previous overexpression and depletion data, the promoter transactivation results showed that RNF4 increased and RNF168 decreased significantly the activity of the *cyclin B1* reporter. In line with previous results, RNF8 expression did not have a substantial effect on the activity of the *cyclin B1* promoter in cycling MCF-7 cells. This ability of RNF168 to specifically repress FOXM1 activity is confirmed by the observation that RNF168 repressed the activity of the wild type (WT), but not a *cyclin B1,* promoter with the mutated FOXM1-binding site (Figure 2b). These results further verified a role for RNF168 in repressing FOXM1 expression and activity.

### RNF168 interacts with FOXM1 in breast cancer cells

**Figure 3:**
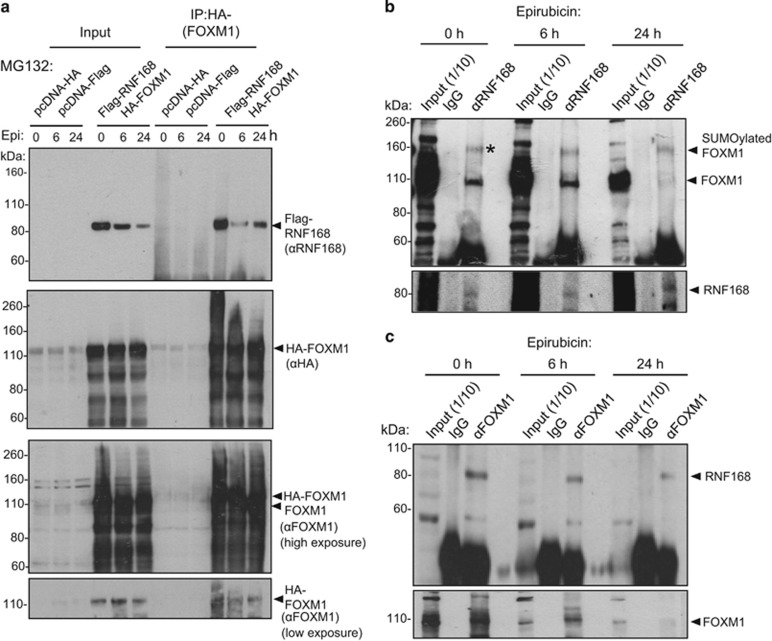
FOXM1 complexes with RNF168 in MCF-7 cells. (**a**) MCF-7 cells co-transfected with either the empty expression vectors, pcDNA3-HA and pcDNA3-Flag, or Flag-RNF168 and HA-FOXM1 were treated with epirubicin (1 μm) for 0, 6 and 24 h. Co-immunoprecipitation (co-IP) was performed with an FOXM1 (αFOXM1) antibody on lysates from these transfected MCF-7 cells pretreated with 10 m MG132; Inputs (1/10 of IP) and IP products with αFOXM1 were resolved on western blot and probed for FOXM1, HA-(FOXM1) and RNF168 expression. Notably, treatment with the proteasome inhibitor MG132 produced high levels of smearing in the western blots, which are undegraded or semi-degraded ubiquitinated protein species. (**b**) Co-immunoprecipitation was performed on MCF-7 cells treated with epirubicin (1 μm) for 0, 6 and 24 h. IP was performed with IgG and an RNF168 (αRNF168) antibody on lysates from these transfected MCF-7 cells. IP products with IgG and a RNF168 antibody (αRNF168) were resolved on western blot and probed for RNF168 and FOXM1. FOXM1* represents a FOXM1 species associated with its SUMOylation.^15^ (**c**) MCF-7 cells were treated with epirubicin (1 μm) for 0, 6 and 24 h. Co-IP was performed with IgG and a FOXM1 antibody (αFOXM1); Inputs (1/10 of IP) and IP products with IgG and a FOXM1 antibody (αFOXM1) were resolved on western blot and probed for RNF168 and FOXM1.

To explore the mechanism by which RNF168 represses FOXM1 expression, we next overexpressed Flag-RNF168 and HA-FOXM1 in MCF-7 cells treated with epirubicin for 0, 6 and 24 h, and studied the interaction of these ectopically expressed proteins by co-immunoprecipitation. The results showed that RNF168 interacted with FOXM1, suggesting that FOXM1 and RNF168 form complexes in breast cancer cells both in the absence and in the presence of epirubicin (Figure 3a). To further confirm this, we carried out co-immunoprecipitation studies on the endogenous RNF168 and FOXM1 protein in MCF-7 cells treated with epirubicin for 0, 6 and 24 h. Consistently, the results showed that RNF168 co-immunoprecipitated with FOXM1 and vice versa, affirming that RNF168 interacts with FOXM1 (Figure 3b).

### Epirubicin induces the association of RNF168 and K48 polyubiquitin chains with FOXM1

**Figure 4:**
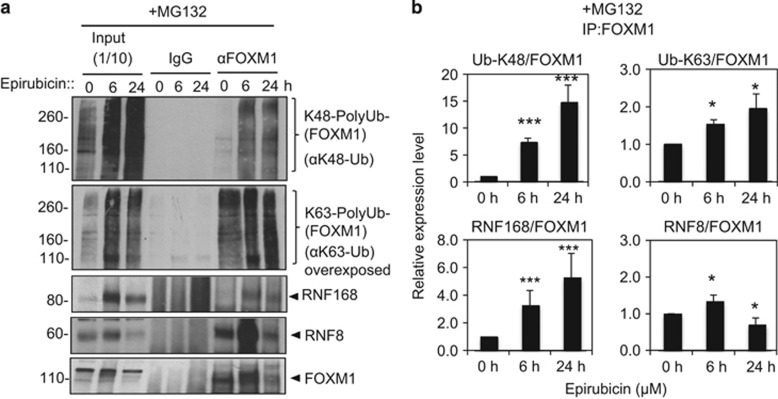
The downregulation of FOXM1 expression by epirubicin is associated with an increase in RNF168 binding and an increase in Lys48-linked polyubiquitin conjugates in MCF-7 cells. (**a**) Protein lysates prepared from MCF-7 and cells at 0, 6 and 24 h following treatment with 1 μm epirubicin were subjected to immunoprecipitation with a FOXM1 antibody (αFOXM1). The input and immunoprecipitates were then analysed by western blot analysis using antibodies against K48-linked ubiquitin, K63-linked ubiquitin, RNF168, RNF8 and FOXM1. Representative co-immunprecipitation results are shown. (**b**) The K48-linked polyubiquitin, K63-linked polyubiquitin, RNF168, RNF8 and FOXM1 images were quantified using ImageJ analysis and plotted against signals at 0 h. The ratios of K48-Ub- and K63-Ub-conjugated FOXM1 relative to FOXM1 precipitates as well as FOXM1 bound RNF168 and RNF8 relative to FOXM1 precipitates were also shown. Data shown represent the mean±s.d. from three independent experiments (*t*-test: 6 h or 24 h versus 0 h epirubicin treatment; *significant *P*<0.05, and ***very significant *P*<0.001). ANOVA was also performed on these data with *post hoc* test.

Given that RNF168 is an E3-Ubiquitin ligase involved in DDR, we proposed that RNF168 binds FOXM1 upon epirubicin treatment and mediates its downregulation through the ubiquitin-proteasome degradation pathway. To test this conjecture, we examined whether RNF168 binding is associated with the induction of FOXM1 ubiquitination in response to epirubicin treatment and studied by co-immunoprecipitation the levels of RNF168, RNF8 and the degradation-related K48-linked polyubiquitin chains associated with FOXM1 in the presence of the proteasome inhibitor MG132 in epirubicin-treated MCF-7 cells (Figure 4). The results revealed that the FOXM1-associated RNF168 protein and K48 ubiquitination chains were at low levels in untreated MCF-7 cells. However, epirubicin treatment caused a significant increase in RNF168 protein and K48 ubiquitination chains co-precipitated with FOXM1 following 6 and 24 h of epirubicin. Conversely, the amounts of FOXM1 and FOXM1 bound RNF8 were at high levels in untreated cells and increased transiently at 6 h before decreasing at 24 h. Quantification of the relative levels of K48-linked polyubiquitin chains, RNF168, and RNF8 associated with FOXM1, revealed that the relative levels of K48-linked polyubiquitin chains and RNF168 associated with FOXM1 actually increased over time upon epirubicin treatment, while the levels of RNF8 bound to FOXM1 increased marginally at 6 h before declining upon epirubicin treatment. Notably, the increase in RNF168 and K48 ubiquitination chains associated with FOXM1 followed similar kinetics and coincided with the decrease in FOXM1 expression, consistent with the notion that RNF168 mediates the ubiquitination and degradation of FOXM1. Despite its expression not correlating directly with FOXM1 ubiquitination and degradation, the fact that RNF8 complexed with FOXM1 also suggests that it may nevertheless have a part in regulating FOXM1 expression, as RNF8 is known to function cooperatively with RNF168 to mediate DDR.^11, 21, 22, 23^

### RNF168 decreases FOXM1 stability in MCF-7 cells and in response to epirubicin treatment

**Figure 5:**
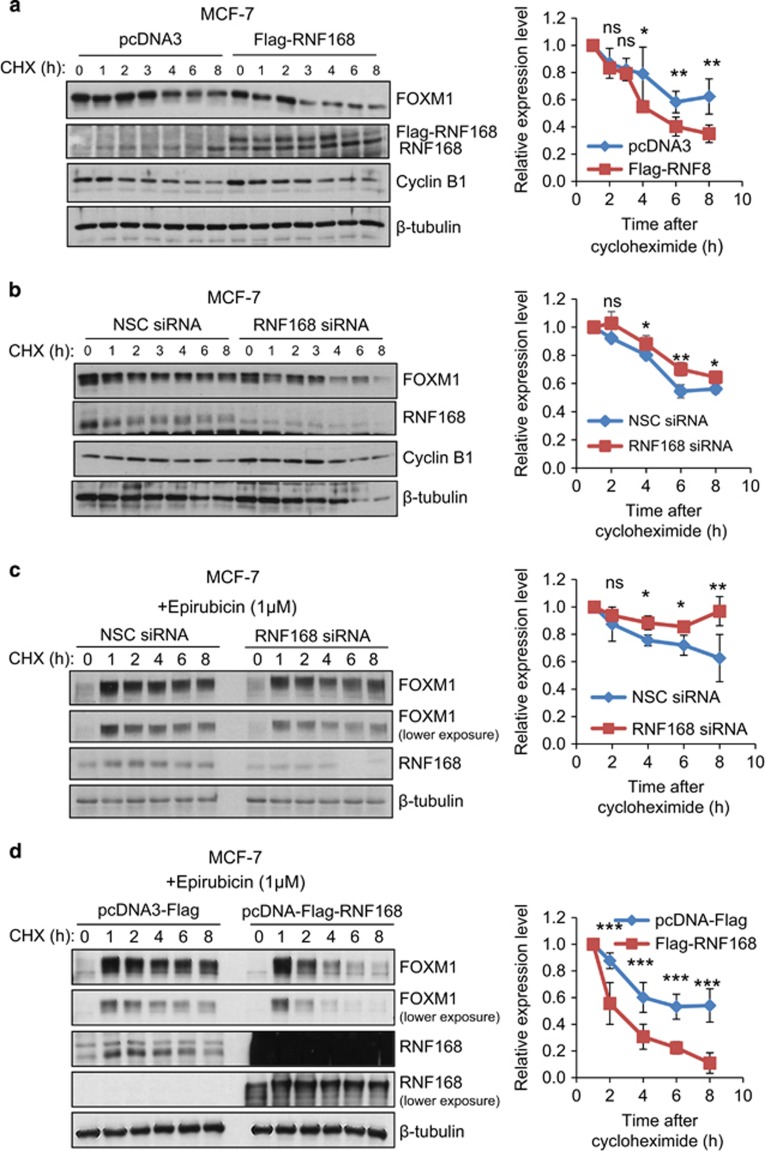
RNF168 enhances the degradation of FOXM1 in untreated and epirubicin-treated MCF-7 cells. (**a**) MCF-7 cells transfected with control pcDNA3 or Flag-RNF168 were treated with CHX, and protein lysates prepared from 0 to 8 h following cyclohexamide treatment. Protein expression levels of FOXM1, RNF168, β-tubulin and cyclin B1 in these MCF-7 lysates were examined by western blotting. (**b**) MCF-7 cells transfected with control NSC siRNA or Smart Pool siRNA targeting RNF168 were treated with CHX, processed and analysed as in (**a**). (**c**) MCF-7 cells transfected with control pcDNA3 or Flag-RNF168 were treated with 1 μm epirubicin for 16 h. Protein lysates prepared from 0 to 8 h following cyclohexamide treatment were processed and analysed as in (**a**). (**d**) MCF-7 cells transfected with control NSC siRNA or Smart Pool siRNA targeting RNF168 were treated with 1 μm epirubicin for 16 h. Protein lysates prepared from 0 to 8 h following cyclohexamide treatment were processed and analysed as in (**a**). Densitometry was used to quantify the FOXM1 and β-tubulin levels from which independent background readings were subtracted. Western blots are representative of three independent experiments. The relative expression levels shown (right panels) are means±s.e.m. of the ratios of FOXM1 to β-tubulin levels relative to those at 0 h, *P⩽0.05, **P⩽0.01, ***P⩽0.001 and ns indicates no significance by student's *t*-test.

To establish a role for RNF168 in regulating FOXM1 protein stability, MCF-7 cells were transfected with either control vector or RNF168, and then treated with the translation inhibitor cycloheximide (CHX) (Figure 5). The results showed that the rate of FOXM1 loss was significantly enhanced in MCF-7 cells transfected with RNF168 compared with cells transfected with the empty vector control (Figure 5a). Conversely, the rates for the decline in FOXM1 levels were reduced in MCF-7 cells transfected with the smart pool siRNA targeting RNF168 compared with the non-silencing control (NSC) siRNA pool (Figure 5b). Notably, the turnover of FOXM1 is significantly but only mildly reduced in MCF-7 cells with RNF168 knockdown. However, this is consistent with our finding that RNF168 expression is low in epirubicin-untreated proliferating MCF-7 cells. We next studied the role of RNF168 in regulating FOXM1 stability in response to epirubicin. Under these conditions, RNF168 overexpression decreased and its depletion increased the stability of FOXM1 compared with the respective controls (Figures 5c and d). Together, these results suggest that RNF168 can modulate the turnover of FOXM1 in untreated breast cancer cells and in response to epirubicin.

### RNF168 links FOXM1 SUMOylation to its degradation

**Figure 6:**
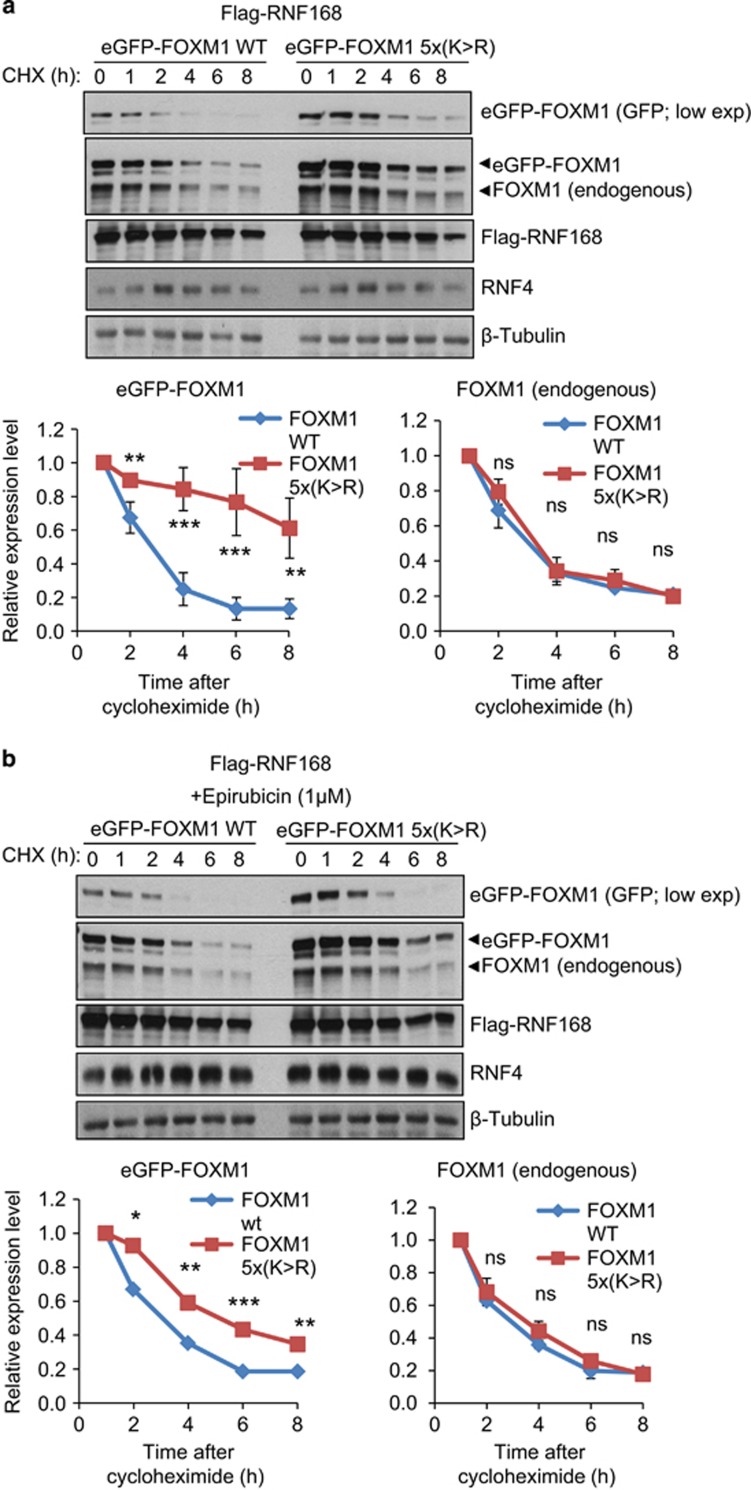
SUMOylation mutant of FOXM1 is resistant to RNF168-mediated degradation. (**a**) Asynchronous MCF-7 cells co-transfected with RNF168 and eGFP-FOXM-1 (WT) or eGFP-FOXM1-5X(K>R) were treated with cyclohexamide and protein lysates prepared from 0 to 8 h following cyclohexamide treatment. Protein expression levels of endogenous FOXM1/eGFP-FOXM1, RNF168 and β-tubulin in these MCF-7 lysates were examined by western blotting. Densitometry was used to quantify the FOXM1/eGFP-FOXM1 and β-tubulin levels from which independent background readings were subtracted. Western blots are representative of three independent experiments. The relative expression levels shown (lower panels) are means ±s.d. of the ratios of FOXM1/eGFP-FOXM1 to β-tubulin levels relative to those at 0 h. (**b**) MCF-7 cells co-transfected with eGFP-FOXM1 (WT) or eGFP-FOXM1-5X(K>R) and Flag-RNF168 were treated with 1 μm epirubicin for 16 h. Protein lysates prepared from 0 to 8 h following cyclohexamide treatment were processed and analysed as in (**a**). Densitometry was used to quantify FOXM1 levels and were normalized to β-tubulin. Western blot representative of three independent experiments (see also Supplementary Figure S1). Statistical significance was determined by Student's *t*-test (*P⩽0.05, **P⩽0.01, ***P⩽0.005; n.s., non-significant) by comparing the densitometry of eGFP-FOXM1 at a particular time.

To examine the hypothesis that RNF168 might link FOXM1 SUMOylation to its ubiquitination and degradation, we co-transfected MCF-7 cells with GFP-FOXM1 (WT) or SUMOylation-defective GFP-FOXM1-5x(K>R) together with RNF168, and compared the ability of RNF168 to enhance the degradation of the WT and the SUMOylation-defective FOXM1 in the presence or absence of epirubicin treatment (Figure 6; Supplementary Figure S1). Compared with endogenous FOXM1 without RNF168 overexpression, overexpression of RNF168 hastened the degradation of both the transfected GFP-FOXM1 (WT) and the endogenous FOXM1. In contrast, we observed that the turnover of the SUMOylation-defective GFP-FOXM1-5x(K>R) was not substantially affected by RNF168 overexpression, suggesting that FOXM1 SUMOylation is required for its targeting by RNF168 for degradation (Figure 6; Supplementary Figure S1).

**Figure 7:**
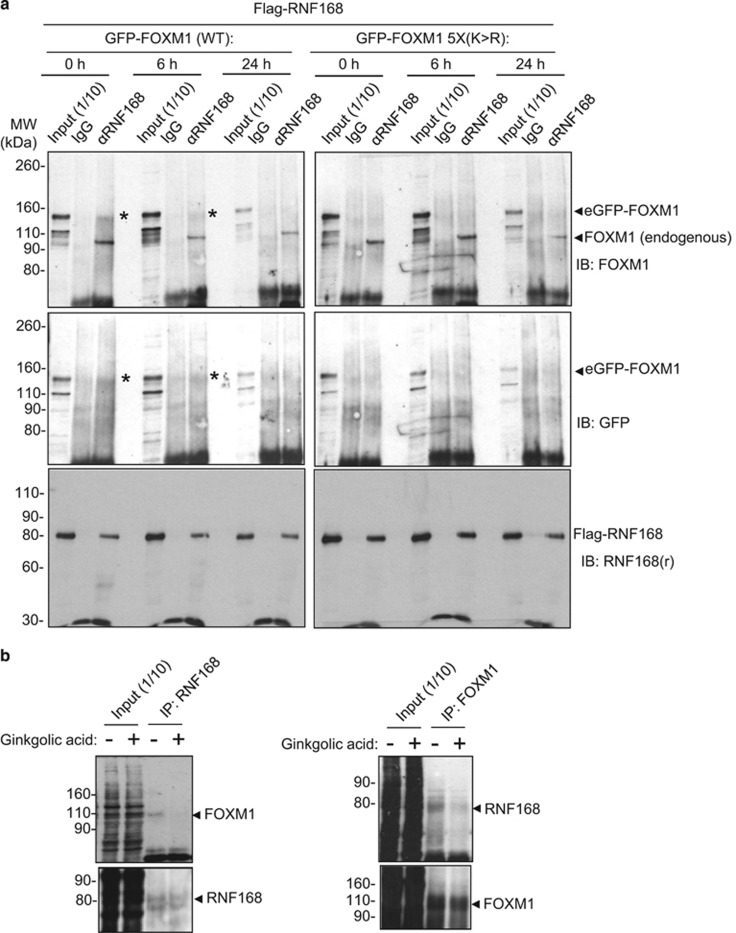
FOXM1 SUMOylation is required for its interaction with RNF168. (**a**) MCF-7 cells were co-transfected with RNF168 and eGFP-FOXM-1 (WT) or eGFP-FOXM1-5X(K>R). Protein lysates prepared from MCF-7 cells at 0, 6 and 24 h following treatment with 1 μm epirubicin were subjected to immunoprecipitation with control antibodies (IgG) or an RNF168 antibody (αRNF168). The input (1/10) and immunoprecipitates were then analysed by western blot analysis using antibodies against FOXM1, GFP and RNF168. Representative co-immunprecipitation results demonstrating that RNF168 only interacts with wild-type FOXM1 but not the SUMOylation mutant FOXM1 are shown. *indicates the position of the eGFP-FOXM1 band. The consistent smear patterns observed for the co-immunoprecipitated FOXM1 might reflect the high degrees of FOXM1 degradation induced by RNF168 binding. (**b**) MCF-7 cells were treated with either vehicle or 10 μm of the protein SUMOylation inhibitor, ginkgolic acid for 4 h. Cells were lysed in RIPA buffer containing 50 mm *N*-ethylmaleimide, and the lysates immunoprecipitated with an RNF168 antibody (left panel) or a FOXM1 antibody (right panel). The input (1/10) and immunoprecipitates were then analysed by western blot analysis using antibodies against FOXM1 and RNF168. The results showed that ginkgolic acid treatment suppresses FOXM1 and RNF168 interaction (see also Supplementary Figure S2).

To further explore the role of RNF168 in coupling FOXM1 SUMOylation to the ubiquitination and degradation pathway, we transfected GFP-FOXM1 (WT) or SUMOylation-defective GFP-FOXM1-5x(K>R) together with Flag-RNF168 into MCF-7 cells, treated with epirubicin for 0, 6 and 24 h, and studied the interaction of these ectopically expressed proteins by co-immunoprecipitation experiments using an anti-Flag antibody (Figure 7a; Supplementary Figure S2). The co-immunoprecipitation results showed that RNF168 interacted with GFP-FOXM1 (WT) but not the SUMOylation-defective GFP-FOXM1-5x(K>R), suggesting that SUMOylation is required for the recruitment of RNF168 to FOXM1 to mediate its ubiquitination and degradation. To confirm this further, we studied the interaction of the endogenous RNF168 with FOXM1 in MCF-7 cells in the absence and presence of ginkgolic acid, a botanical SUMOylation inhibitor, by co-immunoprecipitation (Figure 7b; Supplementary Figure S2). The results showed that RNF168 and FOXM1 interact only in the absence of ginkgolic acid, further suggesting that FOXM1 SUMOylation is required for the recruitment of RNF168.

### RNF168 promotes the direct conjugation of K48-linked polyubiquitin chains to FOXM1

**Figure 8:**
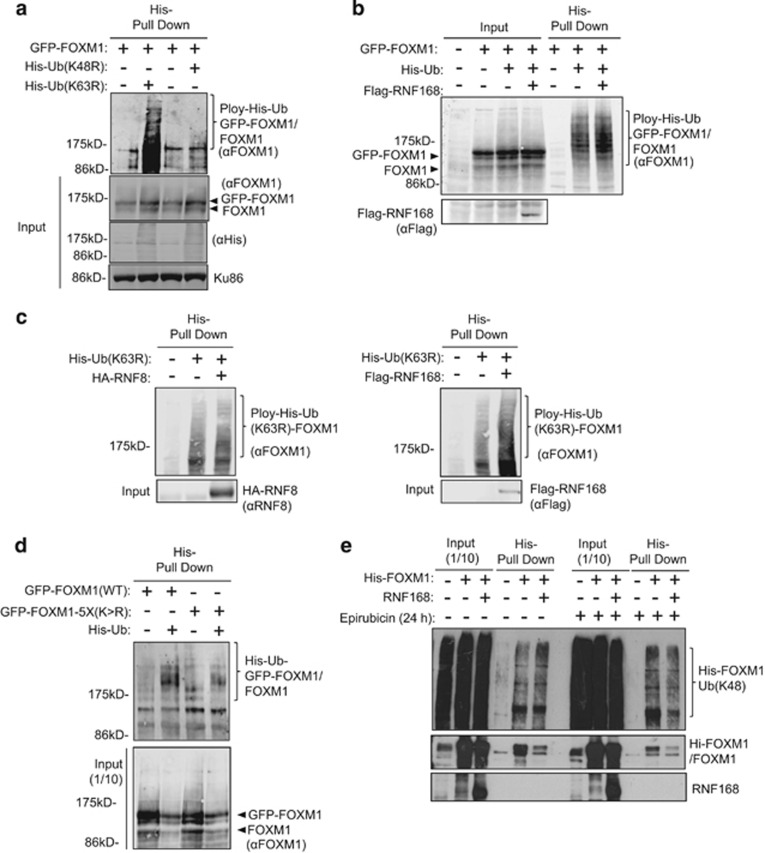
FOXM1 is modified by ubiquitination and its K48-linked polyubiquitin conjugates can be induced by RNF168 and epirubicin. (**a**) 293T cells were co-transfected with eGFP-FOXM1 and empty control expression vector, His-Ubiquitin(K48R) or His-Ubiquitin(K63R). Ubiquitinated proteins were purified using Ni^2+^-column affinity pull-down under denaturing conditions (Guanidinium Chloride). Input and His-tagged Ubiquitin pulled-down proteins were probed for FOXM1 using an anti-FOXM1 antibody. The result showed that higher levels of His-Ub(K63R) were conjugated to FOXM1, suggesting that FOXM1 is primarily covalently linked to K48 polyubiquitin conjugates. (**b**) 293T cells were co-transfected with eGFP-FOXM1 and empty control expression vector or His-Ubiquitin and in the absence or presence of Flag-RNF168. Ubiquitinated proteins were purified using Ni^2+^-column affinity pull-down under denaturing conditions. The input (1/10) and pulled-down proteins were western blotted for FOXM1 expression. The results showed that RNF168 overexpression can further enhance FOXM1 ubiquitination. (**c**) 293T cells were co-transfected with His-Ub(K63R) in the presence or absence of HA-RNF8 or Flag-168. Ubiquitinated proteins were purified under denaturing conditions and probed with FOXM1. The results suggested that RNF168, but not RNF8, can induce FOXM1-K48 polyubiquitination. (**d**) 293T cells were co-transfected with His-Ub and eGFP-FOXM-1 (WT) or eGFP-FOXM1-5X(K>R). His-tagged ubiquitinated proteins were purified under denaturing conditions. The pulled-down His-Ub conjugated proteins were western blotted for FOXM1 expression. The results showed that substantially higher levels of eGFP-FOXM1 WT proteins were pulled down with the His-Ub than the SUMOylation-defective mutant, despite the mutant being the more stable species, suggesting that SUMOylation promotes FOXM1 ubiquitination. (**e**) MCF-7 cells were transfected with His-FOXM1 and Flag-RNF168 or control empty expression vector. Twenty-four hours after transfection, the cells were lysed, the His-tagged FOXM1 purified using Ni^2+^-column affinity pull-down under denaturing conditions and probed with FOXM1, specific K48-linked and K63-linked polyubiquitin antibodies. The results indicated that RNF168 predominantly enhances FOXM1-K48 polyubiquitination in untreated and epirubicin-treated MCF-7 cells.

To investigate whether the polyubiquitin chains are directly conjugated to FOXM1 and to identify the polyubiquitin chains involved, we expressed GFP-FOXM1 in the absence or presence of the His-tagged ubiquitin mutants Ub(K48R) and Ub(K63R) that are defective in forming K48 and K63-linked chains, respectively (Figure 8a). The His-tagged ubiquitinated proteins were then purified using nickel-affinity columns under denaturing conditions in the presence of the proteasome inhibitor MG132 and immunoblotted with an anti-FOXM1 antibody, which detected the ubiquitin-conjugated forms of FOXM1 as smears above the predicted GFP-FOXM1 molecular weight of 160 kDa. The results showed that the GFP-FOXM1 was conjugated to significantly higher levels of Ub(K63R) than Ub(K48R) polymers, suggesting that FOXM1 is linked primarily to K48 polyubiquitin chains, which frequently direct the degradation of target proteins by the 26S proteasome (Figure 8a). We next tested the ability of RNF168 to promote the formation of polyubiquitin chains directly on FOXM1. To this end, His-tagged Ub(WT) was overexpressed with GFP-FOXM1 alone or with GFP-FOXM1 and Flag-RNF168 together in 293T cells, purified under denaturing conditions and probed with an anti-FOXM1 antibody. The results showed that RNF168 overexpression can further increase the amount of ubiquitin chains directly conjugated to FOXM1 (Figure 8b). We then compared the ability of RNF8 and RNF168 to promote the formation of the K48 polyubiquitin chains on FOXM1 by overexpressing His-tagged Ub(K63R) with either HA-RNF8 or Flag-RNF168 (Figure 8c). The results revealed that while the amounts of pulled-down FOXM1 were comparable in control cells and the cells overexpressing RNF8 and RNF168, the amount of His-Ub(K63R) polyubiquitinylated FOXM1 purified was much higher in the RNF168 overexpressing cells, suggesting that RNF168 rather than RNF8 controls the conjugation of K48-linked polyubiquitin chains to FOXM1 in unstimulated cells. Consistent with our earlier findings, the pull-down results also indicated that these directly conjugated polyubiquitin chains required FOXM1 SUMOylation as substantially lower levels of SUMOylation-defective mutant FOXM1 were pulled down by the His-tagged Ubiquitin, compared with the WT FOXM1 (Figure 8d). To further confirm that RNF168 promotes the conjugation of K48-linked polyubiquitin chains to FOXM1, His-tagged FOXM1 was expressed alone or with Flag-RNF168 in MCF-7, and purified under denaturing conditions using nickel-affinity columns in the presence of MG132 (Figure 8e). The polyubiquitin chains covalently conjugated to His-tagged FOXM1 proteins were detected with the anti-K48-linked and anti-K63-linked polyubiquitin chain antibodies. The results revealed that although RNF168 overexpression downregulated FOXM1 expression, the relative levels of K48-linked polyubiquitin chains formed on FOXM1 increased with RNF168 overexpression in both untreated and 24 h epirubicin-treated MCF-7 cells, affirming that RNF168 promotes the conjugation of K48-linked polyubiquitin chains on FOXM1.

### RNF8 is involved in the recruitment of RNF168 and the targeting of SUMOylated FOXM1 for degradation

**Figure 9:**
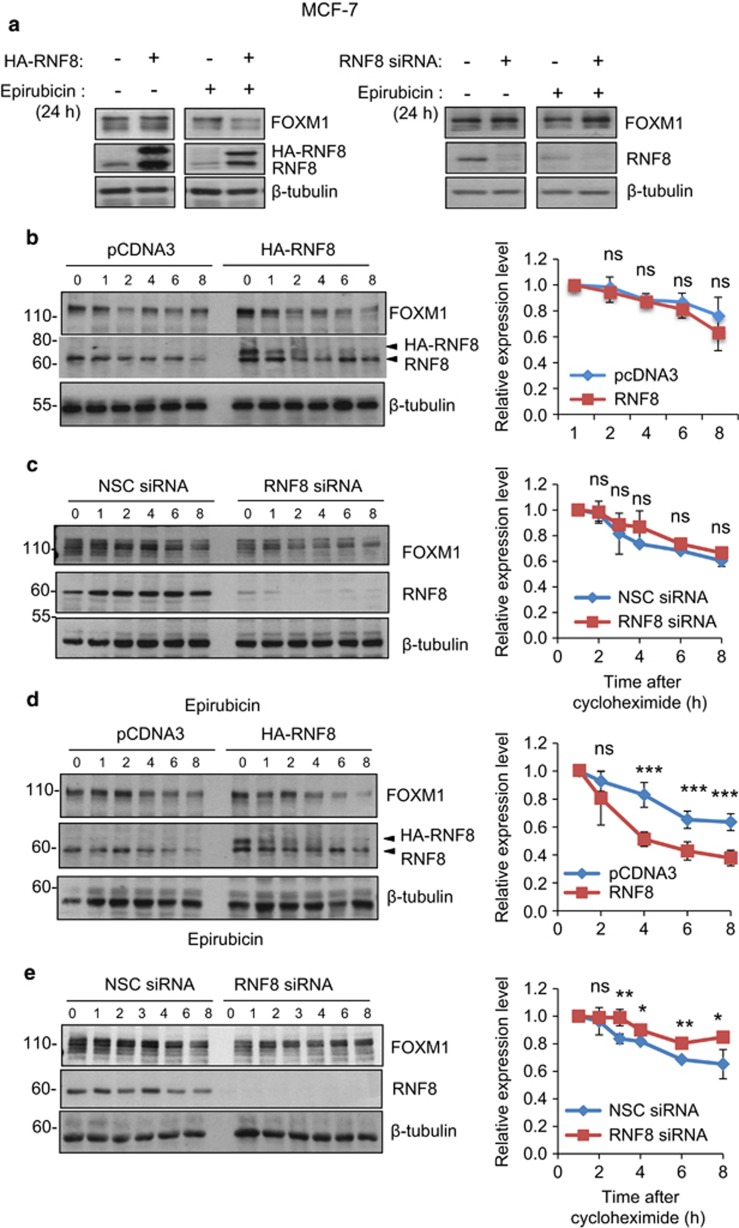
RNF8 enhances the degradation of FOXM1 in epirubicin-treated MCF-7 cells. (**a**) MCF-7 cells were transfected with HA-RNF8 and Smart Pool siRNA targeting RNF8 to study the effects of RNF8 overexpression and depletion, respectively, on FOXM1 expression. Representative western blotting results showing that RNF8 overexpression and depletion only effectively decreases and increases FOXM1 expression in the epirubicin-treated, but not in the untreated, MCF-7 cells. (**b**) MCF-7 cells transfected with control pcDNA3 or HA-RNF8 were treated with CHX, and protein lysates prepared from 0 to 8 h following cyclohexamide treatment. Protein expression levels of FOXM1, RNF8 and β-tubulin in these MCF-7 lysates were examined by western blotting. Densitometry was used to quantify the FOXM1 and β-tubulin levels from which independent background readings were subtracted. Western blots are representative of three independent experiments. The relative expression levels shown (right panels) are means±s.d. of the ratios of FOXM1 to β-tubulin levels relative to those at 0 h. (**c**) MCF-7 cells transfected with control NSC siRNA or Smart Pool siRNA targeting RNF8 were treated with CHX, processed and analysed as in (**b**). (**d**) MCF-7 cells transfected with control pcDNA3 or HA-RNF8 were treated with 1 μm epirubicin for 16 h. Protein lysates prepared from 0 to 8 h following cyclohexamide treatment were processed and analysed as in (**b**). (**e**) MCF-7 cells transfected with control NSC siRNA or Smart Pool siRNA targeting RNF8 were treated with 1 μm epirubicin for 16 h. Protein lysates prepared from 0 to 8 h following cyclohexamide treatment were processed and analysed as in (**b**). Statistical significance was determined by Student's *t*—test (*P⩽0.05, **P⩽0.01, ***P⩽0.005; n.s., non-significant).

**Figure 10:**
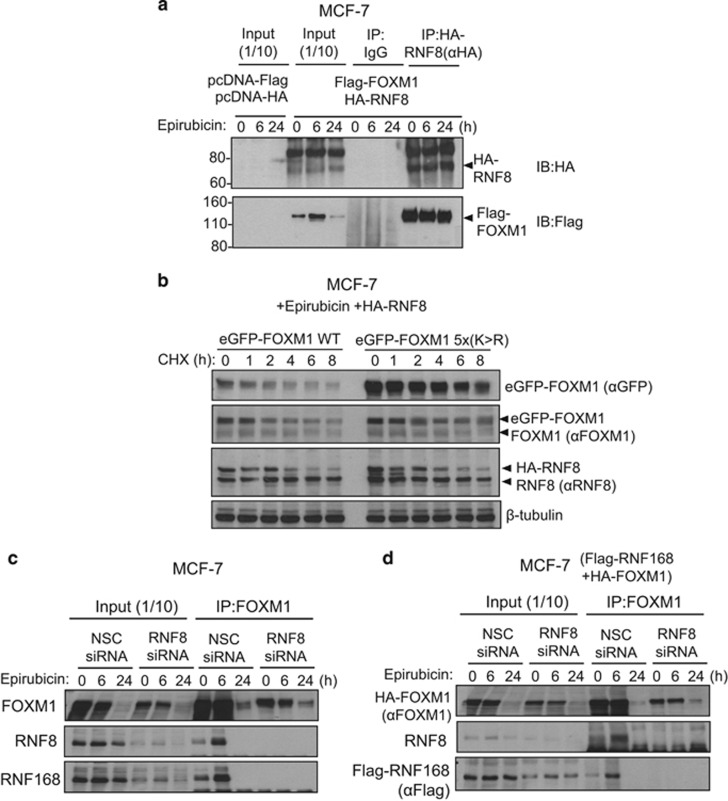
RNF8 binds to FOXM1 and is required for the recruitment of RNF168 and the degradation of SUMOylated FOXM1. (**a**) MCF-7 cells co-transfected with either the empty expression vectors, pcDNA3-HA and pcDNA3-Flag, or HA-RNF8 and Flag-FOXM1 were treated with epirubicin (1 μm) for 0, 6 and 24 h. Co-immunoprecipitation (co-IP) was performed with an FOXM1 (αFOXM1) antibody on lysates from these transfected MCF-7 cells pretreated with 10 μm MG132; Inputs (1/10 of IP) and IP products with αHA were resolved on western blot and probed for Flag-FOXM1 and HA-RNF8 expression. The top band shows a likely post-translationally modified form of RNF8. The results showed that FOXM1 interacts with RNF8. (**b**) MCF-7 cells co-transfected with HA-RNF8, eGFP-FOXM-1 (WT) or eGFP-FOXM1-5X(K>R) were subjected to CHX treatment, and protein lysates prepared from 0 to 8 h following cyclohexamide treatment were subjected to western blot analysis with antibodies against FOXM1, GFP, RNF8 and β-tubulin. Representative western blot results demonstrating that RNF8 targets WT FOXM1 but not the SUMOylation mutant FOXM1 for degradation. (**c**) MCF-7 cells were treated with 1 μm epirubicin for 0, 6 and 24 h. The cells were lysed and immunoprecipitated with IgG or an anti-RNF168 antibody. Inputs (1/10 of IP) and IP products were analysed by western blotting and probed for RNF8, RNF168 and FOXM1 expression. The result further demonstrated that RNF8 is required for the recruitment of RNF168 to FOXM1. (**d**) MCF-7 cells co-transfected with Flag-RNF168, HA-FOXM1 and control NSC siRNA or Smart Pool siRNA targeting RNF8 were treated with 1 μm epirubicin for 0, 6 and 24 h. The cells were lysed and immunoprecipitated with IgG or an anti-RNF168 antibody. Inputs (1/10 of IP) and IP products were analysed by western blotting and probed for RNF8, HA-FOXM1 and Flag-RNF168 expression. The result showed that RNF8 is required for the recruitment of RNF168 to FOXM1.

Although it is evident that RNF168 binds and mediates the ubiquitination of SUMOylated FOXM1, RNF168 possesses only a conventional ubiquitin-interacting motif, but not a SUMO-interacting motif capable of binding specifically to SUMO-conjugated proteins. This evokes the involvement of a STUbL in the initial recognition and ubiquitination of SUMOylated FOXM1. The fact that the DNA damage-related STUbL RNF8 interacts with FOXM1 suggests it might be the STUbL that recognizes SUMOylated FOXM1 and cooperates with RNF168 to mediate FOXM1 ubiquitination and degradation. To test this idea, we examined the effects of overexpression or silencing of RNF8 on the expression and stability of FOXM1 in MCF-7 cells before and after epirubicin treatment (Figure 9). Western blot analysis showed that RNF8 overexpression and silencing decreased and increased the expression of FOXM1 in MCF-7 cells respectively, but only in cells after epirubicin treatment (Figure 9a). This is in agreement with our earlier results that RNF8 overexpression or depletion has little effect on FOXM1 expression in untreated MCF-7 cells (Figure 1a). Consistently, we found that co-expression of RNF8 significantly shortened the half-life of FOXM1, while silencing of RNF8 significantly enhanced the stability of FOXM1 following CHX, in epirubicin-treated MCF-7 cells but not in the untreated cells (Figure 9b; Supplementary Figure S3). To examine whether RNF8 preferentially targets SUMOylated FOXM1 for degradation, we compared the effects of RNF8 on the stability of WT and SUMOylation-defective FOXM1 after CHX treatment. The result showed that co-expression of RNF8 significantly shortened the half-life of WT FOXM1 but not the FOXM1-5X(K>R) mutant following epirubicin treatment, suggesting that RNF8 targets SUMOylated FOXM1 for degradation in response to epirubicin treatment (Figure 10a). Our reverse co-immunoprecipitation experiment (Figure 10b) further confirmed our earlier result that RNF8 interacts with FOXM1 (Figure 4). To further establish our hypothesis, we next investigated whether RNF8 is required for the interaction of RNF168 with FOXM1 (Figure 10c) The result showed that RNF8 is required for the binding of endogenous RNF168 to FOXM1, as depletion of RNF8 using siRNA abolished the interaction between RNF168 and FOXM1 (Figure 10c). To assess this possibility further, we co-expressed Flag-RNF168 with HA-tagged FOXM1 in MCF-7 cells and performed co-immunoprecipitation analysis (Figure 10d). The result confirmed further that RNF8 is required for the interaction between RNF168 and FOXM1. Taken together, our data provide strong evidence that RNF8 is recruited to the SUMOylated FOXM1 to mediate the initial FOXM1 ubiquitination, which results in the recruitment of RNF168 to further enhance FOXM1 ubiquitination and degradation.

### RNF168 and RNF8 restrict cell proliferation and promote epirubicin sensitivity through targeting FOXM1

**Figure 11:**
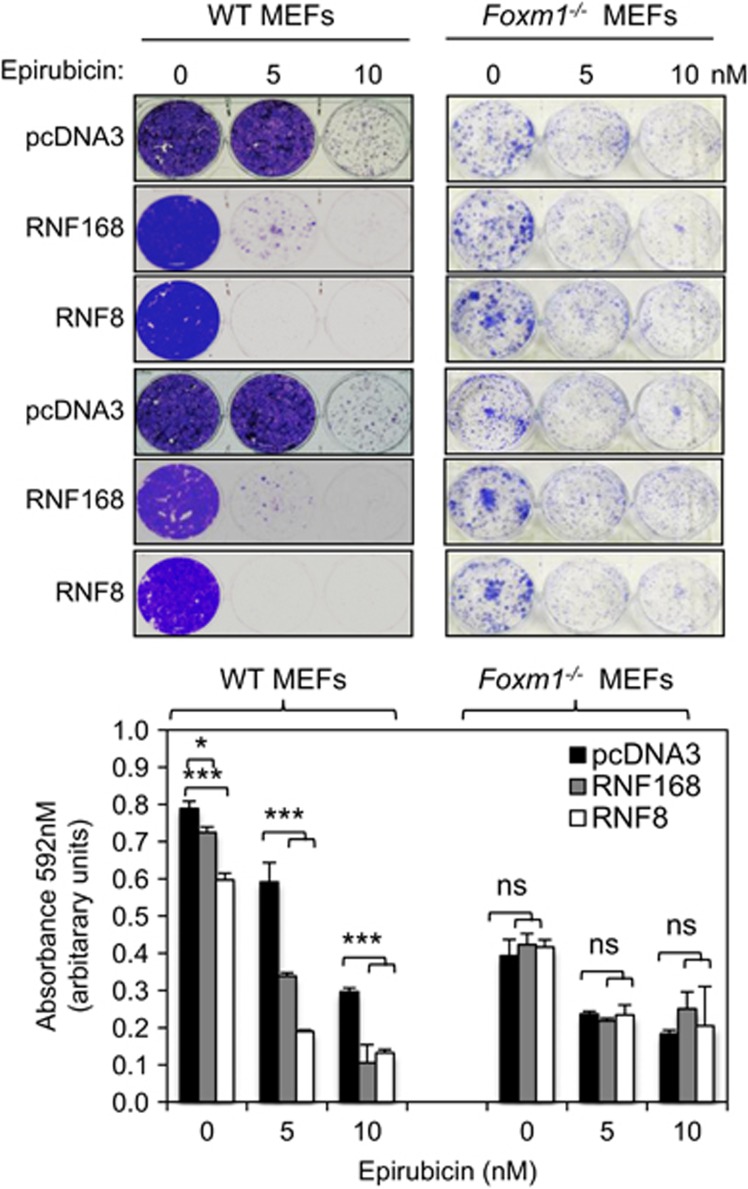
Overexpression of RNF168 and RNF8 reduces clonogenicity in WT but not in *Foxm1*-deficient MEFs. WT and *Foxm1*-deficient MEFs were transfected with the control pcDNA3, HA-RNF8 or Flag-RNF168. Twenty-four hours after transfection, the 2000 cells were seeded in six-well plates, treated with 0, 5 or 10 nm of epirubicn, grown for 15 days and then stained with crystal violet (top panel). The result (bottom panel) represents average of three independent experiments±s.d. Statistical significance was determined by Student's *t*-test (*P⩽0.05, ***P⩽0.005; n.s., non-significant).

FOXM1 has been shown to enhance cancer cell proliferation and protect cells from genotoxic agents by promoting DNA damage repair.^5, 8, 13^ Considering our finding that RNF168 and RNF8 target FOXM1 for degradation in response to epirubicin, we next investigated whether FOXM1 is a target for the anti-proliferative function of RNF168 and RNF8. To this end, we transfected RNF168 and RNF8 into WT and *Foxm1*^*−/−*^ mouse embryo fibroblasts (MEFs), and studied their effects on cell proliferation and in response to epirubicin by clonogenic assay (Figure 11). The results showed that overexpression of RNF168 or RNF8 repressed colony formation and synergized with epirubicin to repress colony formation in WT MEFs, but neither RNF168 nor RNF8 overexpression had any significant effects on the clonogenicity of *Foxm1*^*−/−*^ MEFs in response to epirubicin, suggesting FOXM1 to be a key target of RNF168 and RNF8 in mediating cell proliferative arrest and the genotoxic function of epirubicin. By contrast, there were no significant differences in cell proliferation rates and epirubicin sensitivity in FOXM1-deficient MEFs transfected with vector control, RNF168 and RNF8. These results are in agreement with the notion that RNF168 and RNF8 limit cell proliferation and genotoxic drug resistance thorough promoting FOXM1 ubiquitination and stability (Figure 11).

### Inverse correlation between RNF168 and FOXM1 expression in breast cancer patient samples

**Figure 12:**
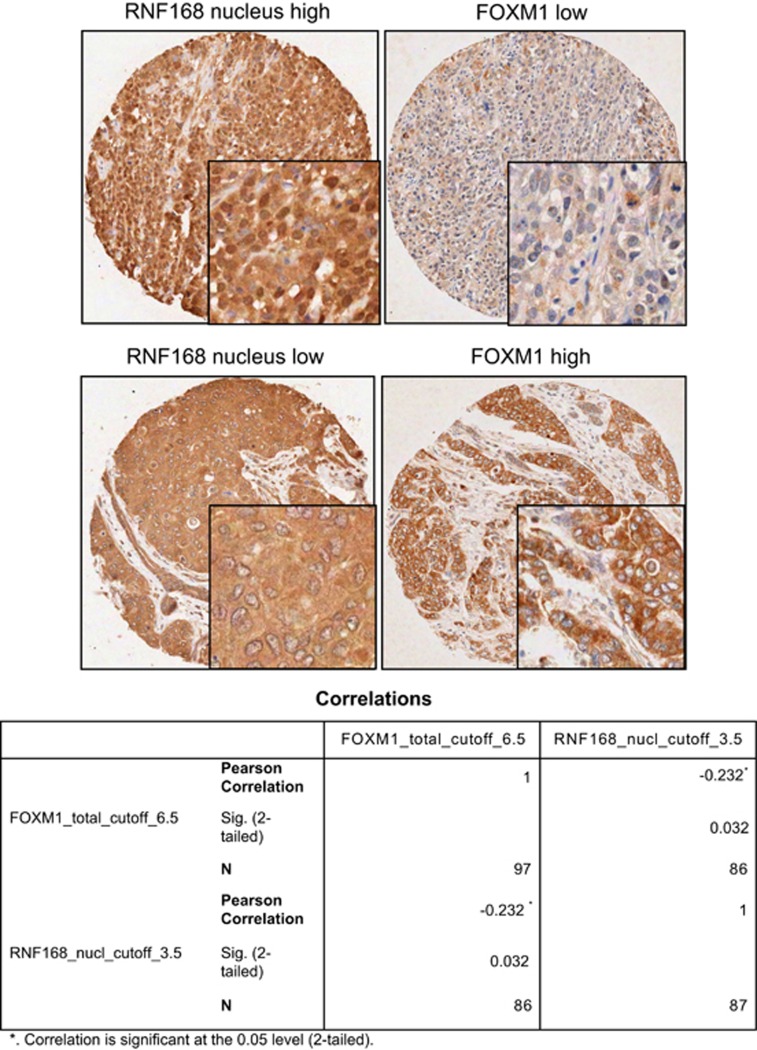
Inverse correlation between FOXM1 and RNF168 expression in breast cancer patients. FOXM1 and RNF168 expression was assessed by immunohistochemistry using tissue microarray constructed from 116 breast cancer patient samples. RNF168 expressed predominantly in the nucleus. Representative staining images of one patient with high FOXM1 and low RNF168 expression and one with low FOXM1 and high RNF168 expression are shown. Images (magnification × 20); Insets (magnification × 100); Inverse correlation between FOXM1 and RNF168 expression was observed. Statistical analysis revealed that RNF168 was significantly correlated with FOXM1 expression (*P*=0.032, Chi-square test; Pearson's correlation coefficient (*r*)=−0.232). Statistical significance (*P⩽0.05).

To establish further the physiological and clinical relevance of the negative regulation of FOXM1 expression by RNF168 in breast cancer, FOXM1 and RNF168 expression was assessed by immunohistochemistry in 116 breast cancer patient samples (Figure 12). RNF168 was predominantly expressed in the nucleus, consistent with its known function as a nuclear ubiquitin E3-ligase enzyme. Immunohistochemical analysis results showed that RNF168 nuclear expression negatively and significantly correlated with FOXM1 expression (Pearson's correlation coefficient: −0.232, *P*=0.032; two-tailed). Consistently, most of the currently known RNF168 functions appear to be restricted to the nucleus. However, there were no significant correlations between RNF168 and other clinicopathological parameters, including PR (progesterone receptor) status, histological type, lymph-node involvement and tumour stage as well as patients' survival. In line with our cell culture data, there were no correlations between RNF8 and FOXM1 expression in these breast cancer patient samples (Supplementary Figure S4), which were obtained not related to any chemotherapy treatment.

## Discussion

FOXM1 has a key role in promoting DNA damage repair and genotoxic agent resistance.^5, 8, 9, 15^ Beside transcriptional control, post-translational regulation has been shown to be another important mode of control for FOXM1 expression, especially in response to DNA damage.^14, 15^ Previous reports have demonstrated that RNF168 can modulate DDR by promoting protein ubiquitination.^11^ In here, we show that RNF168 promotes K48-linked ubiquitination of FOXM1 and targets it for degradation in response to the anticancer DNA-damaging agent epirubicin. Using a SUMOylation-defective mutant FOXM1 and the SUMOylation inhibitor ginkgolic acid, we demonstrated that FOXM1 SUMOylation is required for recruiting RNF168 to FOXM1 to mediate its ubiquitination and degradation. Paradoxically, RNF168 is a ubiquitin (Ub) E3-ligase that possesses Ub-interacting motifs domain but lacks SUMO-interacting motifs,^24^ and therefore, is unlikely to recognize SUMOylated FOXM1 directly in response to epirubicin. In agreement, we found that the recruitment of RNF168 to FOXM1 requires the STUbL RNF8, and the association of FOXM1 with RNF168 is lost in the absence of RNF8 in MCF-7 cells. In a similar manner, RNF168 has also been shown to be recruited to ubiquitinated chromatin proximal to damaged DNA in an RNF8-dependent manner.^24^ Accordingly, RNF168 cannot initiate histone H2A ubiquitination and is recruited only after RNF8-dependent histone ubiquitination in DDR following DSBs.^24^ In line with this, RNF8 is generally observed at DSBs before the recruitment of RNF168.^25^ This cooperation between RNF8/RNF168 in DDR is well documented. For example, RNF168/RNF8 has been shown to mediate K63-linked ubiquitination of the histone H2A and variant H2AX, flanking the DSBs to coordinate DNA repair through recruiting various signalling and repair factors, including BRCA1 and 53BP1.^26^ Moreover, RNF168 also mediates K63-linked ubiquitination of 53BP1 and controls its recruitment to DSBs in DDR.^27^ These pair of E3-ligases have also been shown to function together to enhance DNA repair at uncapped telomeres.^28^ Although the majority of the activities related to the RNF168/RNF8 pathway involve K63-linked ubiquitination and protein recruitment,^26, 27^ we found that RNF8 and RNF168 cooperate to primarily mediate the degradation-associated K48-linked ubiquitination on FOXM1 in response to epirubicin. Consistent with our results, the protein stability of 53BP1, like FOXM1, has also been found to be regulated by RNF8/RNF168-mediated K48-linked ubiquitination for efficient DSB repair.^22^ However, except for 53BP1, few proteins have been identified to be substrates of RNF8/RNF168-dependent K48-linked polyubiquitination.

The activity of ubiquitin E3-ligases is fine-tuned by deubiquitinating enzymes; of which, OTUB1 is one involved in DDR.^29^ We have shown previously that OTUB1 can specifically limit FOXM1 polyubiquitination and its degradation, through its deubiquitinase enzymatic activity.^14^ As a consequence, OTUB1 can antagonize DNA damage-dependent ubiquitination action of RNF8/RNF168 on FOXM1.^29^ Alternatively, OTUB1 can specifically suppress RNF168-dependent polyubiquitination independently of its catalytic activity, through inhibiting UBC13 (also known as UBE2N), the cognate E2 enzyme for RNF168.^29^ Intriguingly, OTUB1 preferentially targets polyubiquitin chains joined by K48-bonds. The fact that OTUB1 specifically targets FOXM1 also supports the fact that FOXM1 is ubiquitinated by K48-linkage chains. It is notable that although FOXM1 SUMOylation is required for the recruitment of RNF8/RNF168 to mediate its degradation, it is possible that these FOXM1 SUMOylation sites are also targets for RNF8/RNF168-mediated ubiquitination, leading to the formation of mixed SUMOylation/ubiquitination chains at these sites. Together, the present and previous findings suggest that RNF8 and RNF168 can be critical limiting factors in the control of FOXM1 expression, particularly upon DNA damage and genotoxic agent treatment.

It is notable that depletion of RNF168 or RNF8 in WT MEFs causes a significant decrease in long-term cell proliferation and clonal renewal in response to epirubicin, but neither RNF168 nor RNF8 knockdown has any appreciable effects on clonogenicity in FOXM1-deficient MEFs. This suggests that both RNF168 and RNF8 target FOXM1 to restrict long-term clonal renewal and to modulate genotoxic agent sensitivity. The high levels of RNF8 and low levels of RNF168 in steady-state untreated MCF-7 breast cancer cells may also explain the inability of RNF8 overexpression or its depletion to modulate FOXM1 expression. However, when the endogenous RNF8 is downregulated and RNF168 upregulated upon DNA damage, RNF8 becomes rate-limiting and can cooperate with RNF168 to enhance FOXM1 ubiquitination and degradation. The physiological relevance of the regulation of FOXM1 by RNF168 is further underscored by the significant inverse correlation between FOXM1 and RNF168 in breast cancer patient samples. The lack of correlations between FOXM1 and RNF8 in clinical samples further supports our observations from tissue culture model that RNF8 expression has little effect on FOXM1 expression in samples not associated with any chemotherapy.

Collectively, these results provide convincing evidence to suggest that the ubiquitin E3-ligases RNF168 and RNF8 couple SUMOylated FOXM1 to its ubiquitination and degradation in response to the genotoxic agent epirubicin. In this way, RNF8 and RNF168 integrate DNA-damage signalling with the transcriptional network of FOXM1 to mediate DDR. These findings expand our understanding on the mechanisms that regulate FOXM1 expression, and have important implications for cancer genotoxic agent treatment and response.

## Materials and methods

### Cell culture, plasmids and transfection reagents

The MCF-7 cell line used originated from the American Type Culture Collection and was acquired through CRUK cell bank (London, UK). MCF-7 cells and MEFs have previously been described.^8, 9^ The eGFP-FOXM1 and pcDNA3-FOXM1 WT and 5x(K>R) mutant expression plasmids have also been described.^15^ The expression vectors for RNF8,^25^ RNF168,^30^ Flag-RNF168^31^ and HA-RNF8 ^32^ have also been described previously. The pcDNA3-RNF4 plasmid was from Prof Ron T. Hay (University of Dundee, Dundee, UK).^33^ The HA-ubiquitin, eGFP-FOXM1(WT) and eGFP-FOXM1-5x(K>R) mutant plasmids have been described.^15^

### Ni-NTA pull-down assays

His-tagged proteins were purified by nickel magnetic agarose beads (Qiagen, Manchester, UK) under denaturing conditions as described.^34^ For details, see also Supplementary Materials and Methods.

### Luciferase reporter assay, gene silencing with siRNAs and measure of FOXM1 protein turnover

Please see Supplementary Materials and Methods.

### Western blotting, co-immunoprecipitation and antibodies

Western blotting was performed on whole-cell extracts by lysing cells in buffer or precipitates in the presence of *N*-ethyl-amide (NEM) (10 mm) (Sigma UK, Poole, UK) as previously described.^9, 35^ See Supplementary Materials and Methods for co-immunoprecipitation and antibodies used.

### Clonogenic and sulphorhodamine-B assays

Clonogenic and sulphorhodamine-B (SRB) assays have been described.^9^ Also see Supplementary Methods and Materials for details.

### Tissue microarray, immunohistochemistry and staining scoring

These reagents and analyses have been described.^9^ For details, see Supplementary Methods and Materials.

### Statistical analysis

All statistics were determined using SPSS 16.0 and Windows XP, Excel (Imperial College London, Software Shop, UK). Also see Supplementary Methods and Materials.
