# Supplementary material for: RNF168 cooperates with RNF8 to mediate FOXM1 ubiquitination and degradation in breast cancer epirubicin treatment
Source: Oncogenesis. 2016 Aug 15;5(8):e252–. doi: 10.1038/oncsis.2016.57 (PMC5007831; doi:10.1038/oncsis.2016.57)
Supplement: Supplementary Materials and Methods [file oncsis201657x1.doc]

**Supplementary Materials and Methods**

***Cell culture, plasmids and transfection reagents***

The MCF-7 cell line used originated from the American Type Culture Collection and was acquired through CRUK cell bank (London, UK). MCF-7 cells and mouse embryo fibroblasts (MEFs) have previously been described. Cells were cultured in DMEM (Sigma-Aldrich, Poole, UK) supplemented with 10% (v/v) FCS and 2 mM glutamine at 37°C. Epirubicin Hydrochloride [2 mg/ml (3.4 mM) in 0.9% Sodium chloride, Medac, Germany] was obtained from Imperial College Healthcare (UK, London).

The pcDNA3-RNF4 plasmid was from Professor Ron T. Hay (University of Dundee, Dundee, UK). The HA-ubiquitin, eGFP-FOXM1(WT) and eGFP-FOXM1-5x(K>R) mutant plasmids have been described. The pcDNA3-RNF168 plasmid was kind gifts from Dr. Daniel Durocher (The Lunenfeld-Tanenbaum Research Institute Mount Sinai Hospital Joseph & Wolf Lebovic Health Complex, Canada). The His-Ub-K48R and -K63R plasmids were from Monsef Benkirane (Institute of Human Genetics - CNRS, Montpellier, France). The His-tagged FOXM1 expression vector was generated from cloning a PCR-generated human FOXM1 cDNA fragment into the pcDNA3.1A/myc-His vector (Invitrogen, Life Technologies, UK). For ubiquitination studies, cells were treated with 10 μM ​MG132 (M7449; Sigma-Aldrich) for 6 h before collection for analysis. Cells were transfected using FuGENE 6 transfection reagent (Promega, Southampton, UK) and XtremeGENE HP reagent (Roche Diagnostics, Welwyn Garden City, UK) as recommended by the manufacturers.

***Western blotting, co-immunoprecipitation and antibodies***

The antibodies against FOXM1 (C-20), β-tubulin (H-235), and Cyclin B1, were purchased from Santa Cruz Biotechnology (Santa Cruz, CA, USA). The K63- and K48-linkage specific polyubiquitin antibodies (D7A11 and 4289) were purchased from Cell Signaling Technology (New England Biolabs Ltd. Hitchin, UK). Antibodies against rabbit RNF8 (ab105362) and sheep RNF168 (AF7217) were obtained from Abcam (Cambridge, UK) and R&D Systems (Abingdon, UK) respectively. Antibodies against Ubiquitin (clone Ubi-1) were from Millipore (U.K.) Limited, (Watford, UK), and Sigma-Aldrich (Poole, UK), respectively. Primary antibodies were detected using horseradish peroxidase-linked anti-mouse or anti-rabbit conjugates as appropriate (Dako, Glostrup, Denmark) and visualized using the ECL detection system (Amersham Biosciences, Pollards Wood, UK).

For co-immunoprecipitation experiments, cell lysates were prepared in IP buffer (1% Nonidet P-40, 150 mM NaCl, 50 mM Tris-HCl [pH 7.4], 10 mM NaF, 1 mM sodium orthovanadate, 10 mM N-ethyl-amide (NEM) and protease inhibitors [Complete protease inhibitor cocktail; Roche, Lewes, UK]) and pre-cleared lysate was immunoprecipitated with the indicated antibodies and protein A/G-sepharose for 2 h. Sepharose beads were then washed in lysis buffer and boiled for 5 min prior to being centrifuged (3500x *g*; 3 min). For cell fractionation MCF-7 cells were lysed in buffer A (10 mM HEPES pH 7.4, 10 mM KCl, 0.1 mM EDTA, 0.1 mM EGTA, 2mM DTT) and protease and phosphatase inhibitors, and incubated for 20 min on ice. NP-40 was added (final concentration 1%v/v) and centrifuged. The supernatant containing the cytoplasmic fraction was then frozen at -70^o^C. The pellet was washed in buffer A and resuspended in buffer B (10 mM HEPES, 10 mM KCl, 0.1mM EDTA, 0.1 mM EGTA, 2 mM DTT, 400 mM NaCl, 1% NP-40), and rotated at 4^o^C for 15 min. Samples were centrifuged at 4^o^C and the supernatent containing the nuclear extract collected and frozen at -70^o^C. Both buffer A and buffer B contained protease and phosphatase inhibitors. Proteins were separated by SDS–PAGE gel electrophoresis, transferred to Hybond-C membranes and immunoblotted with the indicated antibodies.

**Luciferase reporter assay**

Cells were grown in 96-well plates (approximately 5,000 cells/well) and left in an incubator at 37°C 10% CO2 overnight. On the following day, using FuGENE 6 transfection reagent, cells were co-transfected with a *cyclin B1 Luciferase* reporter plasmid, *Renilla* plasmid (pRL-CMV, Promega, UK), transfection efficiency control, and the expression plasmid of interest. For details, please see Supplementary Materials and Methods

***Measure of FOXM1 protein turnover***

The turnover rate of endogenous FOXM1 in MCF-7 cells was determined using cycloheximide (CHX) (01810; Sigma-Aldrich) inhibition of protein synthesis. For details, see Supplementary Materials and Methods

***Gene silencing with siRNAs***

For gene silencing, cells were transiently transfected with siRNA SMARTpool reagents purchased from Thermo Scientific Dharmacon (Lafayette, CO, USA) using Oligofectamine (Invitrogen, Life Technologies Ltd, Paisley, UK) according to the manufacturer’s instructions. siRNAs On-Target plus Smart Pool used were: siRNA FOXM1 (L-009762-00), siRNA RNF168 (L-007152-00), siRNA RNF8 (L-006900-00) and the NSC (non-silencing) control siRNA (D-001810-10-05).

***Ni-NTA pull-down assays***

Ni-NTA pull down assays were performed by incubating Ni-NTA magnetic agarose beads (Qiagen, Manchester, UK) with appropriate HeLa cell lysates overnight in buffer A containing 10 mM imidazole, 6 M guanidinium chloride, 100 mM NaH_2_PO4 (pH. 8), 0.05% Tween20. The Ni-NTA beads were washed five times with buffer A and then 3 times with buffer B consisting of 8 M urea, 100 mM NaH_2_PO4 (pH 5.9), 0.05% Tween20 containing 10 mM imidazole, 20 mM Tris (pH 7.5), 150 mM NaCl, 2 mM EDTA and 0.05% (v/v) Triton X-100. Magnetic agarose-bound proteins were separated by SDS-PAGE and Western blots were visualized by ECL.

***Measure of FOXM1 protein turnover***

The turnover rate of endogenous FOXM1 in MCF-7 cells was determined using cycloheximide (CHX) (01810; Sigma-Aldrich) inhibition of protein synthesis. MCF-7 were transiently transfected with either pcDNA empty expression vector, or wild-type (WT) or mutant FOXM1. Twenty-four hours after transfection CHX was added to the culture media to a final concentration of 80 μg/ml. Cells were harvested at indicated time points, and equal amounts of cell lysates were subjected to SDS-PAGE and analysed by immunoblotting (IB).

***Clonogenic Assay***

A total 2,000 cells were seeded into six-well plates and incubated overnight. The cells were then treated for 48 h with varying concentrations of epirubicin. The drug was removed and surviving cells were left to form colonies. After 14 days of incubation, colonies were fixed with 4% Paraformaldehyde for fifteen minutes at room temperature and then washed with phosphate buffered saline. 0.5% crystal violet was used to stain the fixed cells for thirty minutes, following which the plates were washed with tap water. Plates were then left to dry overnight. Quantification was achieved by solubilising dye with 33% acetic acid and the absorbance was measured at 492nm using a microplate reader (Sunrise, Tecan, CA, USA).

***Sulphorhodamine-B (SRB) assay***

Clonogenic and Sulphorhodamine-B (SRB) assays have been described {Khongkow, 2014 #946}. Also see Supplementary Methods and Materials for details.

Cells were seeded in 96-well plates (~5,000 cells/well) and left to grow at 37°C with 10% CO_2_ overnight. On the following day, the cells were treated with the drug of interest. To harvest, cells were fixed by adding 100 µl of ice-cold 40% (w/v) trichloroacetic acid (TCA, Sigma-Aldrich, UK) to each well and incubated at 4°C for 1 h. Plates were rinsed 3 times with slow running water before being stained with 100 µl of 0.4% (w/v) Sulphorhodamine-B (SRB, Sigma-Aldrich, UK) in 1% (v/v) acetic acid (VWR International, UK), followed by incubation for 1 h at room temperature. Following incubation, the plates were washed with 1% (v/v) acetic acid and left to be dry overnight. For measurement, 100 µl of 10 mM Tris-base (VWR International, UK) was used to dissolve SRB from cells in a shaker for 30 min. Optical density (OD) was read at 492 nm using a microtitre plate reader (Sunrise, Tecan, Reading, UK). Results were normalised to untreated or control wells. This assay was used for measuring protein content, the difference between each condition was assumed to be equivalent to a difference of the number of cells.

***Tissue Microarray***

One hundred and thirty-three cases of breast cancer diagnosed between the years 1992 to 2001 with clinical follow up data were retrieved from the records of the Department of Pathology, Queen Mary Hospital of Hong Kong, with approval by the Institutional Review Board of the University of Hong Kong. Histological sections of all cases were reviewed by the pathologist, the representative paraffin tumour blocks chosen as donor block for each case and the selected areas marked for construction of tissue microarray (TMA) blocks. A total of 116 could be assessed and scored for FOXM1, RNF168 and RNF8 staining. The expression pattern and subcellular localization were correlated with various clinicopathological data including ER, PR status, age, histological grade, histological type, clinical stage, lymph node metastasis as well as survival time.

***Immunohistochemistry***

The TMA sections were deparaffinized and rehydrated by incubation with xylene and decreasing concentrations of ethanol. Citrate buffer (0.01M, pH 6.0) was used for antigen retrieval. The slides were immersed into 3% H_2_O_2_/methanol for 10 min at room temperature to quench endogenous peroxidase. After rinsing in 0.05% Tween in PBS (PBST) twice, FOXM1 (c-20; Santa Cruz, USA), RNF8 (ab105362; Abcam) and RNF168 (AF7217; R&D Systems) specific antibodies diluted at 1:1100, 1:50 and 1:50 respectively, were added to each section and incubated at 4°C overnight. The slides were then washed in PBST and incubated with DAKO EnVision+System-HRP-labelled Polymer Anti-Rabbit at room temperature in dark for 30 min. After washing, Chromogen DAB/substrate reagent was added onto the slides and the slides incubated for a further 6 minutes. Finally, the slides were dehydrated and mounted. Aperio ScanScope ® system (Aperio technology, USA) was used to visualize and assess for protein expression.

***Staining scoring***

The stained TMA slides were scanned by ScanScope scanners and individual stained TMA spots were assessed in computer screen with the use of Aperio’s image viewer, ImageScope. To avoid subjectivity in evaluation, the intensities and percentages of the staining were scored by two independent individuals in a semi-quantitative way and average was taken. For each case, a final score was obtained by multiplying the score of intensity with the score of percentage, 12 being the maximum final score.

***Statistical analysis***

The correlation between FOXM1 and RNF168 or RNF8 expression in TMA was assessed by bivariate Pearson Correlation analysis. The correlation between OTUB1 expression and patients’ survival was estimated by Kaplan-Meier estimation and compared by Log-rank test. Multivariate analysis was done by Cox-regression model. Where appropriate a two-tailed independent sample t-test was performed to analyse significance. *P* value less than 0.05 was considered to be statistically significant.

***Luciferase reporter assay***

Cells were grown in 96-well plates (approximately 5,000 cells/well) and left in an incubator at 37°C 10% CO2 overnight. On the following day, using FuGENE 6 transfection reagent, cells were co-transfected with a *cyclin B1 Luciferase* reporter plasmid {*Renilla* plasmid (pRL-CMV, Promega, UK), transfection efficiency control, and the expression plasmid of interest. For details, please see Supplementary Materials and Methods

To harvest, after 24 h of transfection, cells were washed with PBS before being lysed with 100 µl/well of 1x Steady lite plus reagent (Perkin Elmer, UK) and incubated for 15 min in a dark environment. Lysates were transferred to a luciferase plate (Perkin Elmer, UK) where the emission of Luciferase-derived light can be measured using a PHERAstar Plus microplate reader (BMG Labtech, Aylesbury, UK). Then, 25 μl of the renilla substrate mix (1% (w/v) coelenterazine (Lux Biotechnology, UK), 0.04 M EDTA, 0.5 M HEPES pH 7.8) was added to each well and incubated 20 min in the dark. The presence of EDTA inhibited the Luciferase enzyme. The luminescence light from Renilla was measured using the PHERAstar Plus microplate reader.

The Luciferase reading was normalised to the renilla reading, well by well. All measurements were performed in six replicates.
