# Supplementary material for: RNF168 cooperates with RNF8 to mediate FOXM1 ubiquitination and degradation in breast cancer epirubicin treatment
Source: Oncogenesis. 2016 Aug 15;5(8):e252–. doi: 10.1038/oncsis.2016.57 (PMC5007831; doi:10.1038/oncsis.2016.57)
Supplement: Supplementary Figure S1 [file oncsis201657x2.ppt]

## Slide 1
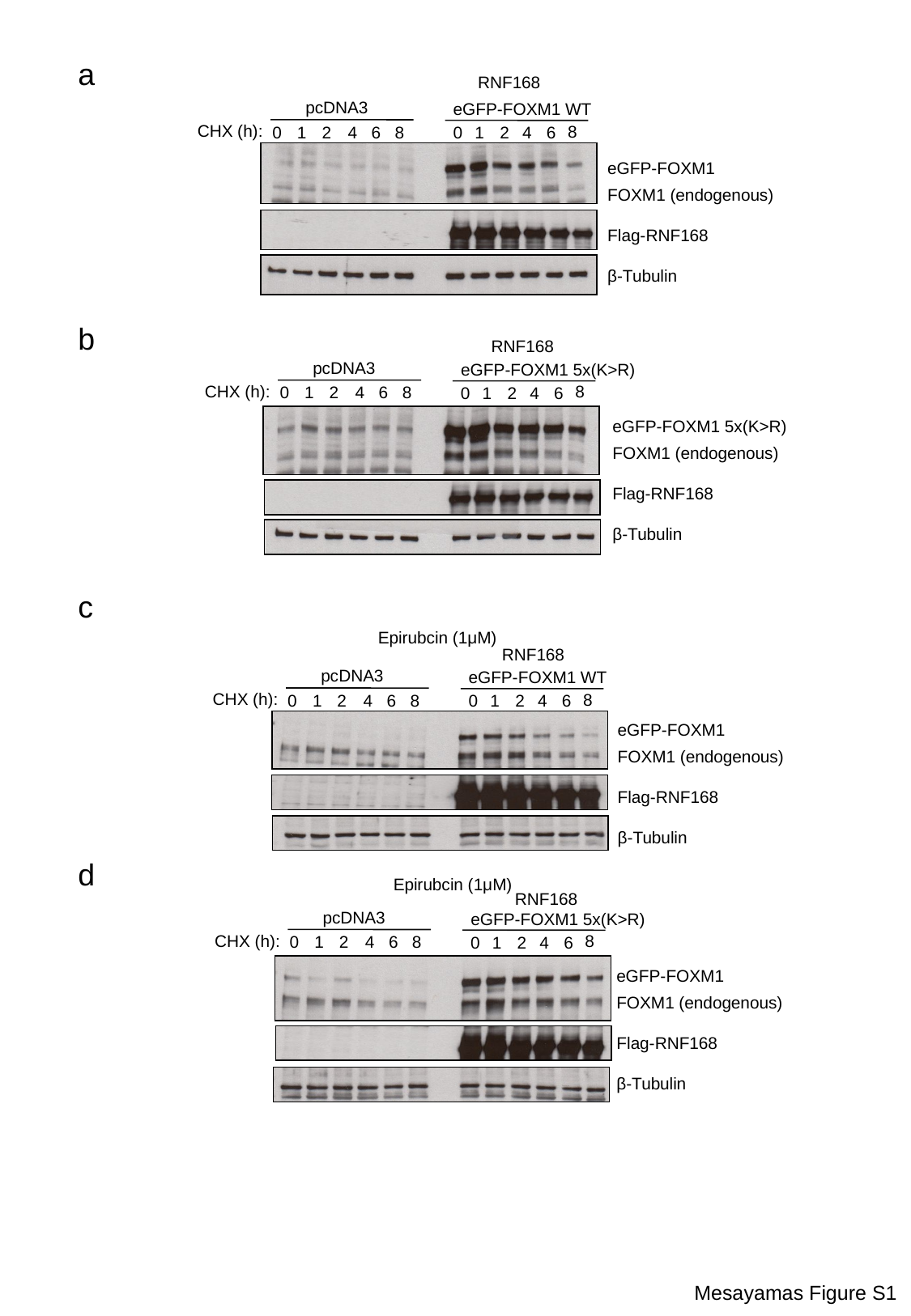

a
RNF168
pcDNA3
eGFP-FOXM1 WT
CHX (h):
8
0
1
2
4
6
8
0
1
2
4
6
eGFP-FOXM1
FOXM1 (endogenous)
Flag-RNF168
β-Tubulin
b
RNF168
pcDNA3
eGFP-FOXM1 5x(K>R)
CHX (h):
8
0
1
2
4
6
8
0
1
2
4
6
eGFP-FOXM1 5x(K>R)
FOXM1 (endogenous)
Flag-RNF168
β-Tubulin
c
Epirubcin (1μM)
RNF168
pcDNA3
eGFP-FOXM1 WT
CHX (h):
8
0
1
2
4
6
8
0
1
2
4
6
eGFP-FOXM1
FOXM1 (endogenous)
Flag-RNF168
β-Tubulin
d
Epirubcin (1μM)
RNF168
pcDNA3
eGFP-FOXM1 5x(K>R)
CHX (h):
8
0
1
2
4
6
8
0
1
2
4
6
eGFP-FOXM1
FOXM1 (endogenous)
Flag-RNF168
β-Tubulin
Mesayamas Figure S1
