# Supplementary material for: RNF168 cooperates with RNF8 to mediate FOXM1 ubiquitination and degradation in breast cancer epirubicin treatment
Source: Oncogenesis. 2016 Aug 15;5(8):e252–. doi: 10.1038/oncsis.2016.57 (PMC5007831; doi:10.1038/oncsis.2016.57)
Supplement: Supplementary Figure S2 [file oncsis201657x3.ppt]

## Slide 1
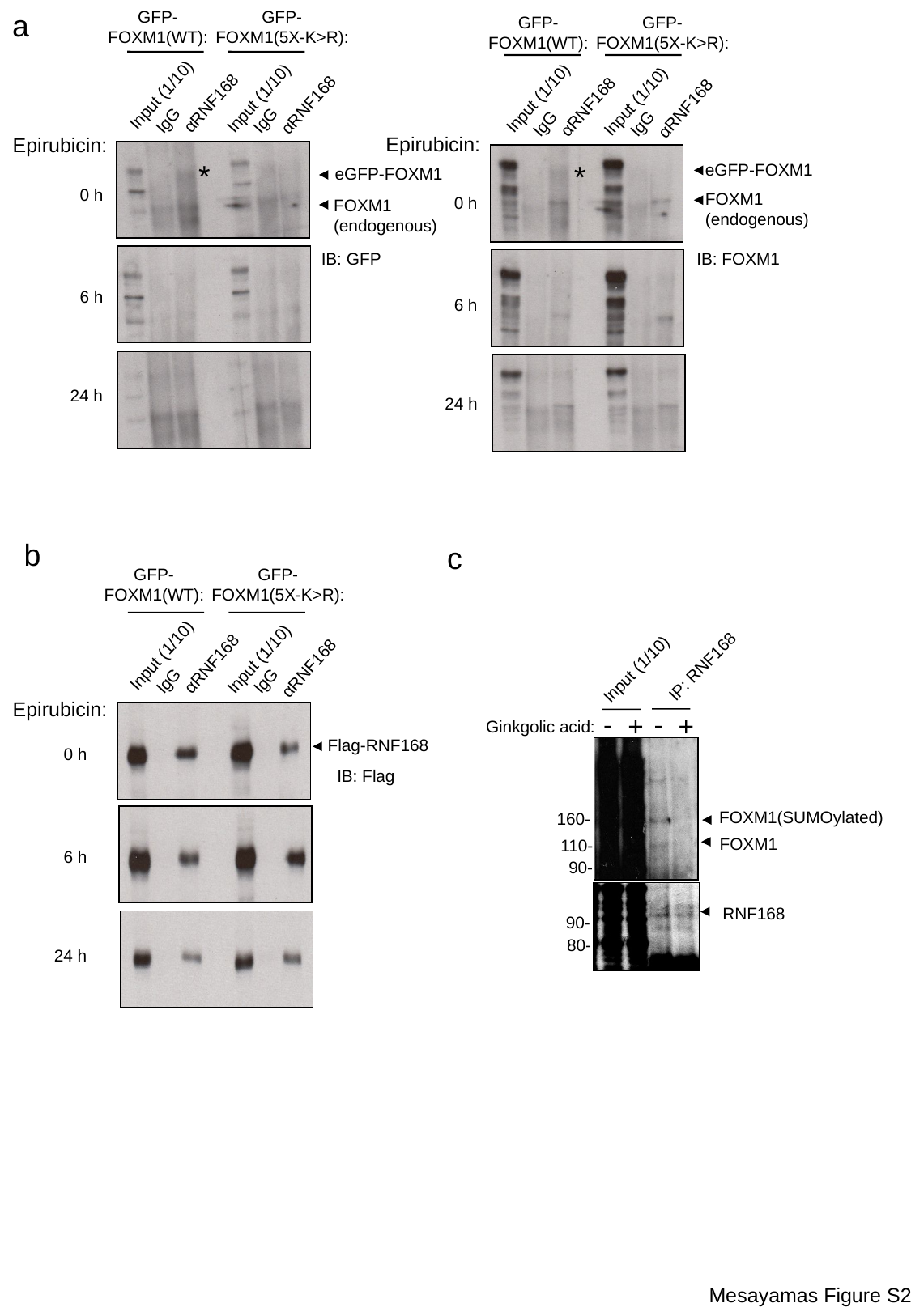

a
GFP-
FOXM1(WT):
GFP-
FOXM1(5X-K>R):
GFP-
FOXM1(WT):
GFP-
FOXM1(5X-K>R):
Input (1/10)
Input (1/10)
Input (1/10)
Input (1/10)
αRNF168
αRNF168
αRNF168
αRNF168
IgG
IgG
IgG
IgG
Epirubicin:
Epirubicin:
*
*
eGFP-FOXM1
eGFP-FOXM1
0 h
FOXM1
(endogenous)
0 h
FOXM1
(endogenous)
IB: GFP
IB: FOXM1
6 h
6 h
24 h
24 h
b
c
GFP-
FOXM1(WT):
GFP-
FOXM1(5X-K>R):
Input (1/10)
Input (1/10)
αRNF168
IP: RNF168
αRNF168
Input (1/10)
IgG
IgG
Epirubicin:
-
-
+
+
Ginkgolic acid:
Flag-RNF168
0 h
IB: Flag
FOXM1(SUMOylated)
160-
FOXM1
110-
6 h
90-
RNF168
90-
80-
24 h
Mesayamas Figure S2
