# Supplementary material for: RNF168 cooperates with RNF8 to mediate FOXM1 ubiquitination and degradation in breast cancer epirubicin treatment
Source: Oncogenesis. 2016 Aug 15;5(8):e252–. doi: 10.1038/oncsis.2016.57 (PMC5007831; doi:10.1038/oncsis.2016.57)
Supplement: Supplementary Figure S3 [file oncsis201657x4.ppt]

## Slide 1
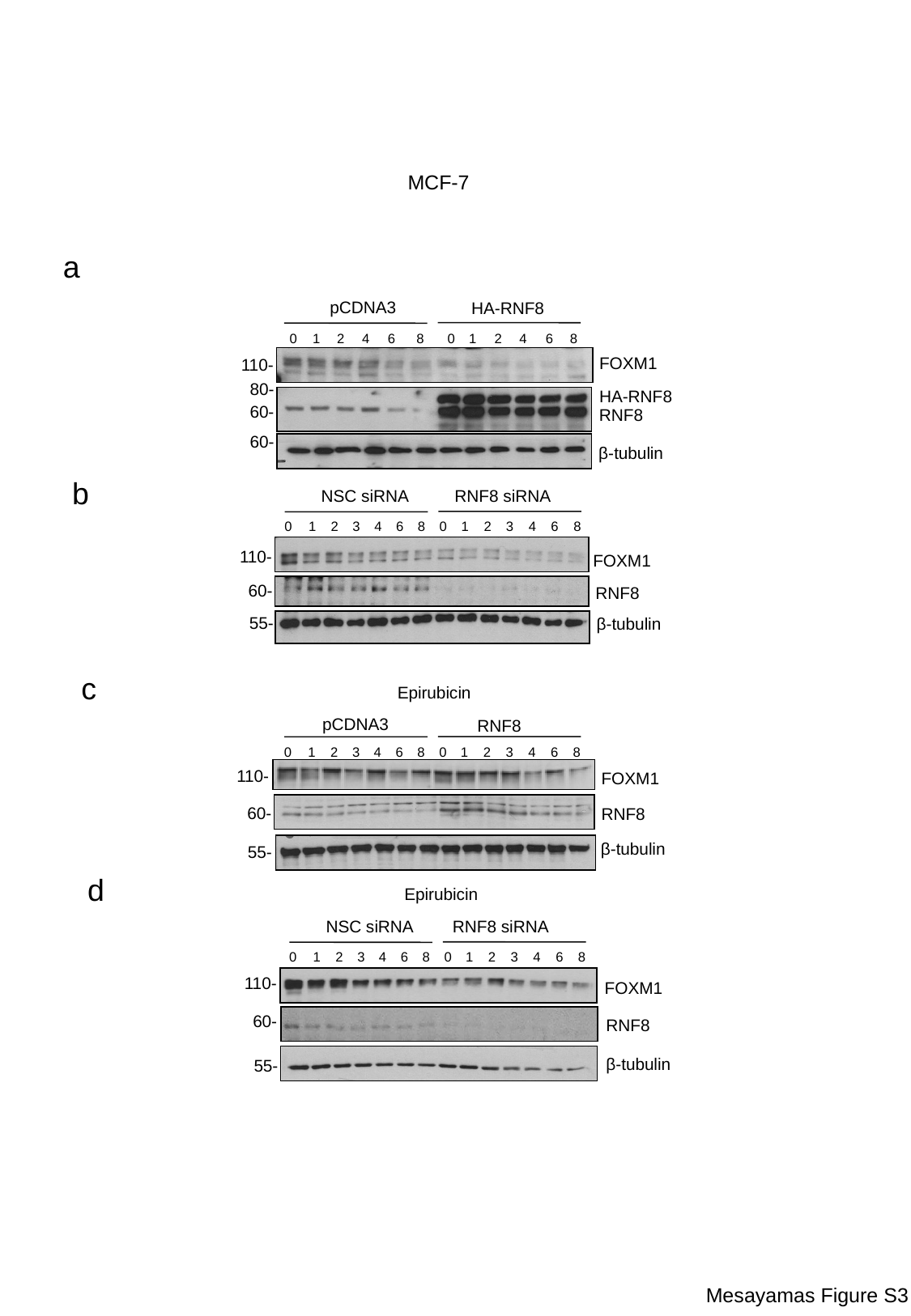

MCF-7
a
pCDNA3
HA-RNF8
0
1
2
4
6
8
0
1
2
4
6
8
FOXM1
110-
80-
HA-RNF8
60-
RNF8
60-
β-tubulin
b
NSC siRNA
RNF8 siRNA
0
1
2
3
4
6
8
0
1
2
3
4
6
8
110-
FOXM1
60-
RNF8
55-
β-tubulin
c
Epirubicin
pCDNA3
RNF8
0
1
2
3
4
6
8
0
1
2
3
4
6
8
110-
FOXM1
60-
RNF8
β-tubulin
55-
d
Epirubicin
NSC siRNA
RNF8 siRNA
0
1
2
3
4
6
8
0
1
2
3
4
6
8
110-
FOXM1
60-
RNF8
β-tubulin
55-
Mesayamas Figure S3
