# Supplementary material for: RNF168 cooperates with RNF8 to mediate FOXM1 ubiquitination and degradation in breast cancer epirubicin treatment
Source: Oncogenesis. 2016 Aug 15;5(8):e252–. doi: 10.1038/oncsis.2016.57 (PMC5007831; doi:10.1038/oncsis.2016.57)
Supplement: Supplementary Figure S4 [file oncsis201657x5.ppt]

## Slide 1
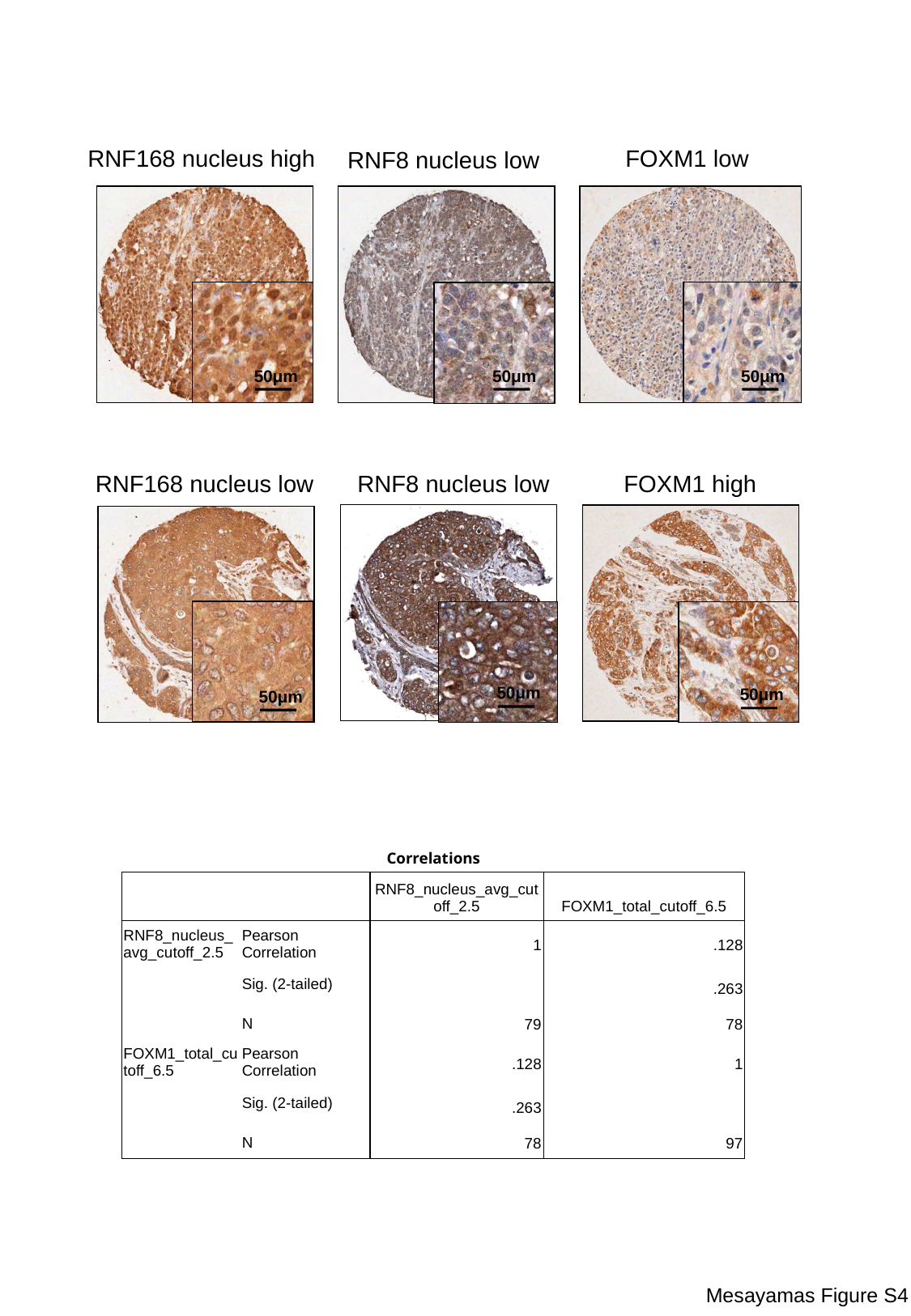

RNF168 nucleus high
FOXM1 low
RNF8 nucleus low
50μm
50μm
50μm
RNF168 nucleus low
RNF8 nucleus low
FOXM1 high
50μm
50μm
50μm
| Correlations | | | |
| --- | --- | --- | --- |
| | | RNF8\_nucleus\_avg\_cutoff\_2.5 | FOXM1\_total\_cutoff\_6.5 |
| RNF8\_nucleus\_avg\_cutoff\_2.5 | Pearson Correlation | 1 | .128 |
| | Sig. (2-tailed) | | .263 |
| | N | 79 | 78 |
| FOXM1\_total\_cutoff\_6.5 | Pearson Correlation | .128 | 1 |
| | Sig. (2-tailed) | .263 | |
| | N | 78 | 97 |
Mesayamas Figure S4
