# Supplementary material for: RNF168 cooperates with RNF8 to mediate FOXM1 ubiquitination and degradation in breast cancer epirubicin treatment
Source: Oncogenesis. 2016 Aug 15;5(8):e252–. doi: 10.1038/oncsis.2016.57 (PMC5007831; doi:10.1038/oncsis.2016.57)
Supplement: Supplementary Figure Legends [file oncsis201657x6.doc]

**Supplementary Figure Legends**

**Supplementary Figure S1. SUMOylation mutant of FOXM1 is resistant to RNF168-mediated degradation.** Asynchronous MCF-7 cells either untransfected or co-transfected with RNF168 and **(a and c)** eGFP-FOXM-1 (WT) or **(b and d)** eGFP-FOXM1 5X(K>R) were treated with cyclohexamide and protein lysates prepared from 0-8 h following cyclohexamide treatment. Protein expression levels of endogenous FOXM1/eGFP-FOXM1, RNF168, and β-Tubulin in these MCF-7 lysates were examined by western blotting. In the top panels, the MCF-7 cells were untreated, but the MCF-7 cells in the lower panels were treated with 1μM epiribicin for 16 h. The results showed that RNF168 overexpression increases the degradation rates of wild-type FOXM1 (endogenous and ectopic) but not the SUMOylation mutatnt FOXM1 5X(K>R).

**Supplementary Figure S2. FOXM1 SUMOylation is required for its interaction with RNF168. a)** MCF-7 cells were co-transfected with RNF168 and eGFP-FOXM-1 (WT) or eGFP-FOXM1 5X(K>R). Protein lysates prepared from MCF-7 cells at 0, 6 and 24 h following treatment with 1 μM epirubicin were subjects to immunoprecipitation with control antibodies (IgG) or an RNF168 antibody (αRNF168). The Input (1/10) and immunoprecipitates were then analysed by western blot analysis using antibodies against FOXM1 and GFP. The Representative co-immunprecipitation results demonstrating that RNF168 only interacts wild-type FOXM1 but not the SUMOylation mutant FOXM1 are shown. **b)** The Input (1/10) and immunoprecipitates were also probed for RNF168 expression. **c)** MCF-7 cells were treated with either vehicle or 10 μM of the protein SUMOylation inhibitor, ginkgolic acid for 4 h. Cells were lysed in RIPA buffer containing 50 mM *N*-ethylmaleimide, and the lysates immunoprecipitated with an RNF168 antibody. The Input (1/10) and immunoprecipitates were then analysed by western blot analysis using antibodies against FOXM1 and RNF168. The results showed that ginkgolic acid treatment suppresses FOXM1 and RNF168 interaction.

**Supplementary Figure S3. RNF8 enhances the degradation of FOXM1 in epirubicin-treated MCF-7 cells.**

**a)** MCF-7 cells transfected with control pcDNA3 or HA-RNF8 were treated with 1μM epiribicin for 16 h, and protein lysates prepared from 0-8 h following cyclohexamide treatment. Protein expression levels of FOXM1, RNF8, and β-Tubulin in these MCF-7 lysates were examined by western blotting. **b)** MCF-7 cells transfected with control NSC siRNA or Smart Pool siRNA targeting RNF8 were treated with cycloheximide, processed and analysed as in **(a)**. **c)** MCF-7 cells transfected with control pcDNA3 or HA-RNF8 were treated with 1 μM epirubicin for 16 h. Protein lysates prepared from 0-8 h following cyclohexamide treatment were processed and analysed as in **(a)**. **d)** MCF-7 cells transfected with control NSC siRNA or Smart Pool siRNA targeting RNF8 were treated with 1 μM epirubicin for 16 h. Protein lysates prepared from 0-8 h following cyclohexamide treatment were processed and analysed as in **(a).**

**Supplementary Figure S4. No correlation between FOXM1 and RNF8 expression in breast cancer patients. (a)** FOXM1 and RNF8 expression was assessed by immunohistochemistry using tissue-microarray constructed from 116 breast cancer patient samples. RNF8 expressed predominantly in the nucleus. Representative staining images of one patient with high FOXM1, low RNF168 and low RNF8 expression and one with low FOXM1, high RNF168 and and low RNF8 expression are shown (same patients as shown in Figure 12. Images (magnification 20 X); Insets (magnification 100 X) No correlation between FOXM1 and RNF8 expression was observed. Statistical analysis revealed that RNF8 were not correlated with FOXM1 expression (*P= 0.263,* Chi-Square test; Pearson's correlation coefficient (r)=0.128).
